# Associations of PD-1 and PD-L1 gene polymorphisms with cancer risk: a meta-analysis based on 50 studies

**DOI:** 10.18632/aging.205689

**Published:** 2024-03-27

**Authors:** Maoquan Yang, Yan Liu, Shuangshuang Zheng, Peizhen Geng, Tianhao He, Linan Lu, Yikuan Feng, Qiqi Jiang

**Affiliations:** 1 School of Clinical Medicine, Shandong Second Medical University, Weifang 261042, Shandong, China; 2 Department of Gastroenterology, Weifang People’s Hospital, The First Affiliated Hospital of Shandong Second Medical University, Kuiwen, Weifang 261000, Shandong, China; 3 Department of Health, Weifang People’s Hospital, The First Affiliated Hospital of Shandong Second Medical University, Kuiwen, Weifang 261000, Shandong, China

**Keywords:** PD-1, PD-L1, polymorphism, cancer risk, meta-analysis

## Abstract

Programmed death-1 and its ligand-1 (PD-1/PD-L1), immune checkpoints proteins, play a crucial role in anti-tumor responses. A large number of studies have evaluated the relationships of PD-1/PD-L1 polymorphisms with risk of cancer, but evidence for the associations remains inconsistent. Therefore, we performed a meta-analysis to examine the associations between PD-1/PD-L1 single nucleotide polymorphisms (SNPs) and cancer predisposition. Results showed that PD-1.3 and PD-L1 rs17718883 were significantly correlated with overall cancer risk. PD-1.5 was prominently linked with cervical cancer (CC), non-small cell lung cancer (NSCLC), TC (thyroid cancer), brain tumor, AML (acute myelocytic leukemia) and UCC (urothelial cell carcinoma) risk, PD-1.9 with breast cancer (BC), AML, esophageal cancer (EC) and ovarian cancer (OC) risk, and PD-1.3 with colorectal cancer (CRC) and BCC (basal cell carcinoma) risk. PD-1.1 polymorphism slightly elevated BC and OC susceptibility, whereas the rs4143815 variant notably decreased the risk of gastric cancer (GC), hepatocellular carcinoma (HCC) and OC, but increased the risk of BC. PD-1.6 was closely linked with AML risk, PD-L1 rs2890658 with NSCLC, HCC and BC risk, rs17718883 with HCC and GC risk, rs10815225 with GC risk, and rs2297136 with NSCLC and HCC risk. Interestingly, the rs7421861, rs10815225, and rs10815225 markedly reduced cancer susceptibility among Asians. The rs7421861 polymrophism decreased cancer risk among Caucasians, rather than the rs10815225 elevated cancer risk. Our results supported that PD-1 and PD-L1 SNPs were dramatically correlated with cancer risk.

## INTRODUCTION

Cancer has become the second leading cause of death with high prevalence and mortality rate, greatly influencing the public health and global economy worldwide [1]. According to the latest statistics, there were 19.3 million new cancer cases and almost 10 million cancer mortalities, and an estimated 28.4 million cases is predicated to occur in 2040 [2]. Carcinogenesis is a complex multifactorial and multiple-step process involving gene-environment interactions [3]. It has been reported that many factors, including heavy alcohol consumption, lack of exercise, high-calorie diet, smoking, chemical dyes, and genetic factor, may contribute to the occurrence and progression of cancer [4–7]. Accumulative genome-wide association studies (GWAS) have been optimized to search for potential genetic with cancer risk [8]. The programmed cell death protein 1 and its ligands (PD-1/PD-Ls) gene has attracted extensive attention for its critical role in the maintenance of immune tolerance [9].

Growing evidence has shown that the immune system plays a key role in resisting and eliminating cancer cells. T lymphocytes are considered to be main cells in anti-tumor immune response, and take part in the occurrence and development of cancer [10, 11]. The activation and proliferation of T lymphocytes depend on the stimulatory and inhibitory signals from CD28/B7 family members [12]. Therefore, single nucleotide polymorphisms (SNPs) of immune response-related genes that regulate T lymphocyte function and alter immune status may contribute to the pathogenesis and progression of various cancers [13].

As a member of the CD28/B7 superfamily with 50-55 KDa, PD-1 is mainly expressed by activated T cells that are responsible for the negative regulation of T cell activation and peripheral tolerance [14, 15]. It is encoded by programmed cell death-1 (PDCD1) gene localized on chromosome 2q27.3. Interaction between PD-1 and its ligands PD-L1 can suppress the activation and proliferation of T-lymphocytes, and production of cytokine through triggering a vital signaling pathway, resulting in apoptosis [16, 17]. PD-Ls are commonly expressed on the non-lymphoid organs, and several antigen-presenting cells (APCs), such as macrophages, dendritic cells (DCs), lymphocytes [18]. PD-Ls have been reported to be highly expressed in various carcinomas, including breast cancer (BC), gastric cancer (GC), colorectal cancer (CRC), and cervical cancer (CC) [19–22]. T cell immune response. Over-expression of PD-L1 in malignancies induces T cell failure via PD-1/PD-L1 signaling pathway, allowing tumor cells to evade host immune surveillance and T cell immune attack, thus leading to poor clinical prognosis and cancer recurrence [23]. In fact, blockade of PD-1/PD-L1 axis strengthens an efficient anti-tumor T cell responses and a better control of tumor [24]. Clinical trials of immunotherapy on antibody-mediated PD-1 blockade are in progress in patients with all kinds of cancers [25].

Recent studies have investigated the potential associations between PD-1/PD-L1 polymorphism and cancer risk, but the results are still controversial. For example, Emma L et al. first verified that the PD-1.5 variation was markedly correlated with lower CC risk [26]. Zhang et al. found that PD-1.3, PD-1.1, PD-1 rs7421861, PD-L1 rs17718883, and rs4143815 were dramatically related to the cancer predisposition [27]. A study reported that PD-1.5, PD-1.3 and PDL-1 rs4143815 remarkably decreased cancer risk, while PD-1 rs7421861 notably enhanced the cancer risk [28]. Dong et al. discovered that PD-1.5 was strongly related to decreased cancer risk [29]. In 2019, Zou et al. proved an evident relationship of PD-L1 rs4143815 with increased risk of GC, bladder cancer and hepatocellular carcinoma [30]. Therefore, we conducted this meta-analysis to validate the relationships of PD-1.5 (rs2227981), PD-1.9 (rs2227982), PD-1.3 (rs11568821), PD-1.1 (rs36084323), PD-1 rs7421861, PD-L1 rs4143815, PD-1.6 (rs10204525), PD-L1 rs2890658, rs10815225, rs17718883, and rs2297136 gene polymorphisms with risk of cancer.

## RESULTS

### Literature search and screening

**Figure 1:**
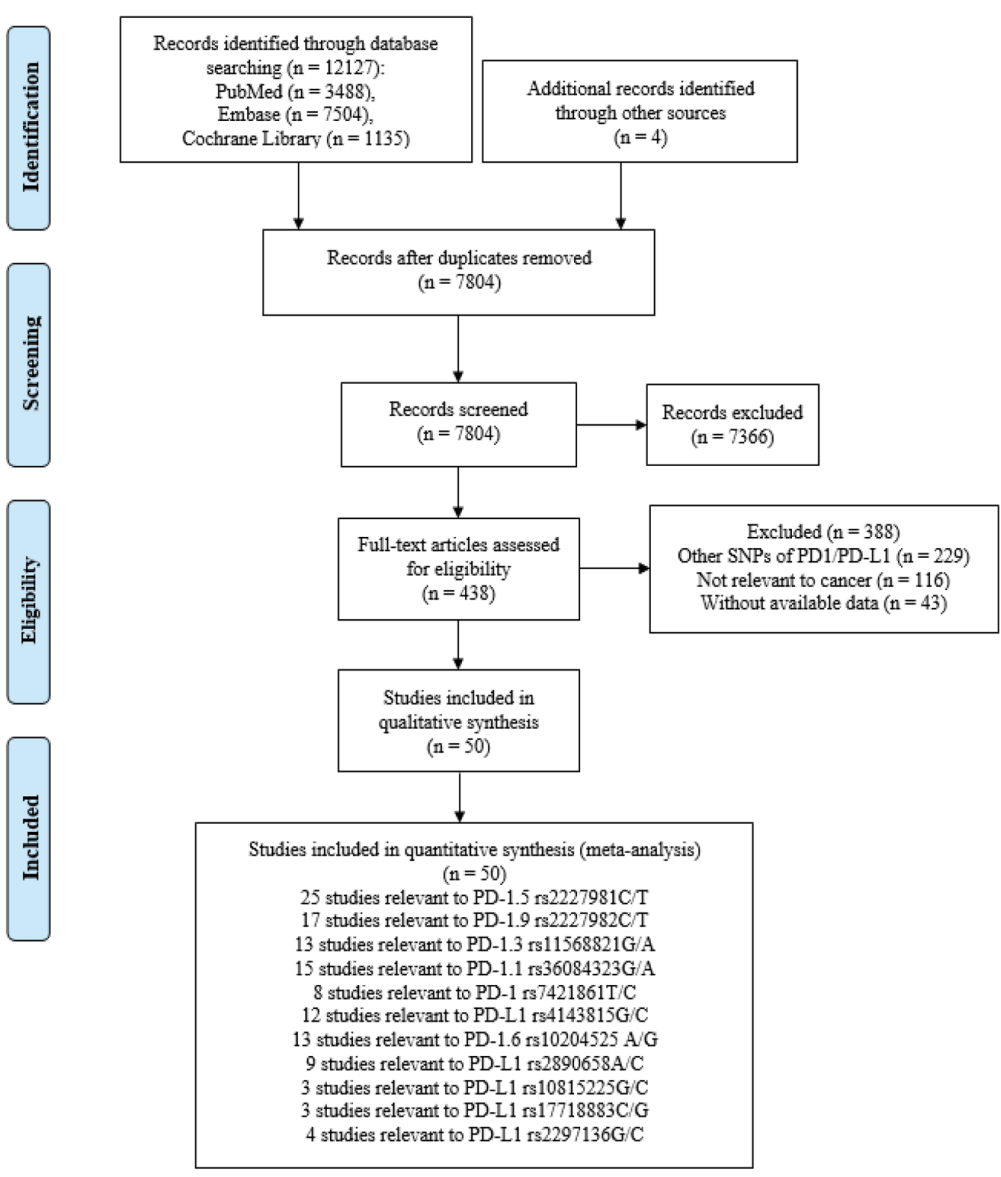
Flow chart of search strategy and study selection.

The systematic search initially yielded 10081 potentially relevant articles through PubMed (n = 3488), Embase (n = 7504), and Cochrane Library (n = 1135) databases, and 4 additional records [31–34] were retrieved from other sources. After the elimination of 4325 duplicate references, 7804 additional publications were removed by screening the abstract and title. Of these, 6383 articles were reviews, meta-analysis, editorials, letters, and conference abstracts, while 983 articles were involved in animal or vitro studies. After careful review of the full texts, 388 studies were further excluded due to the following reasons: 229 studies focused on other SNPs of PD1/PD-L1, 116 studies were not related to cancer and 43 studies lacked of available data. Finally, 50 eligible publications were qualified for this meta-analysis [13, 26, 31–78]. The flow diagram showed the detailed literature search and selection process in Figure 1.

### Characteristics and quality of studies

A total of 50 eligible articles embodying 122 studies were enrolled in the present analysis, which included 25 studies for PD-1.5, 17 studies for PD-1.9, 13 studies for PD-1.3, 15 studies for PD-1.1, 8 studies for rs7421861, 12 studies for PD-L1 rs4143815, 13 studies for PD-1.6, 9 studies for rs2890658, 3 studies for rs10815225, 3 studies for rs17718883, and 4 studies for rs2297136. Among these studies, twenty-six studies were conducted in China, thirteen in Iran, three in Turkey, two in Poland, two in Japan, one in Sweden, one in Czech, one in Brazil, and one in Saudi Arabian. Five studies were from Caucasian population, and forty-five studies were from Asian population. The control group of 19 studies were based on population, and 31 studies on hospital. Twenty-three reported gastrointestinal neoplasms including esophageal cancer (EC), gastric cancer (GC), hepatocellular carcinoma (HCC), and colorectal cancer (CRC). Ten studies investigated cervical cancer (CC), breast cancer (BC) and ovarian cancer (OC), six covered non-small cell lung cancer (NSCLC), and eleven studies involved other types of cancer, such as TC (thyroid cancer), Brain tumor, Melanoma, HNSCC (head and neck squamous cell carcinoma), Myeloma, BCC (basal cell carcinoma), Leukemia, RCC (renal cell carcinoma), AML (acute myelocytic leukemia) and UCC (urothelial cell carcinoma). Detailed characteristics of these studies are illustrated in Table 1 and Supplementary Table 1. All articles are of high quality because of the Newcastle-Ottawa Scale (NOS) score no less than 6 in Supplementary Table 2.

**Table 1:** Characteristics of the included studies in our meta-analysis.

**Author**	**Year**	**Country**	**Ethnicity**	**Type of cancer**	**Sample size case/control**	**Genotyping** **methods**	**Source of control**	**NOS**
Emma L [26]	2010	Sweden	Caucasian	CC	1306/811	PCR-RFLP	PB	7
Haghshenas [35]	2011	Iran	Asian	BC	443/328	PCR-RFLP	PB	8
Hua Z [36]	2011	Chian	Asian	BC	490/ 512	PCR-RFLP	PB	8
Bayram S [37]	2012	Turkey	Asian	HCC	236/236	PCR–RFLP	HB	7
Mojtahedi Z [38]	2012	Iran	Asian	CRC	200/200	PCR–RFLP	PB	7
Li [39]	2013	China	Asian	HCC	271/318	TIANamp	PB	8
Yousefi AR [40]	2013	Iran	Asian	CRC	80/100	PCR-RFLP	HB	6
Savabkar S [41]	2013	Iran	Asian	GC	122/166	PCR–RFLP	HB	7
Wang WP [42]	2013	China	Asian	GC	205/393	Sequencing	HB	6
Chen YB [43]	2014	China	Asian	NSCLC	293/293	PCR-RFLP	HB	7
Qiu H [44]	2014	China	Asian	EC	629/686	PCR-LDR	HB	7
Yin L [45]	2014	China	Asian	NSCLC	324/330	PCR	PB	8
Cheng SS [46]	2015	China	Asian	NSCLC	288/300	PCR-RFLP	HB	6
Ge J [47]	2015	China	Asian	CRC	596/620	TaqMan	HB	7
Ma Y [48]	2015	China	Asian	NSCLC	528/600	PCR-RFLP	PB	9
Tang WF [49]	2015	China	Asian	EC	330/608	PCR-LDR	HB	7
Li XF [50]	2016	China	Asian	CC	256/250	PCR-RFLP	PB	8
Ren HT [51]	2016	China	Asian	BC	560/583	MassARRAY	PB	8
Haghshenas [52]	2016	Iran	Asian	TC	105/160	PCR-RFLP	PB	8
Zhou RM [53]	2016	China	Asian	EC	584/585	PCR-LDR	PB	9
Li Q [31]	2016	China	Asian	GC	101/141	PCR	HB	7
Tao [54]	2016	China	Asian	GC	350/500	Sequencing	HB	6
Du [55]	2017	China	Asian	NSCLC	320/199	Sequencing	HB	7
Zhou RM [56]	2017	China	Asian	EC	575/577	PCR-LDR	PB	9
Jahromi [57]	2017	Iran	Asian	Brain tumor	152/150	PCR-RFLP	PB	8
Li Y [58]	2017	China	Asian	OC	620/620	PCR-LDR	HB	7
Tan D [59]	2017	China	Asian	OC	164/170	qRT-PCR	PB	9
Tang WF [60]	2017	China	Asian	EC	1063/1677	PCR-LDR	HB	7
Cheng SG [32]	2017	China	Asian	HCC	123/141	PCR	HB	8
Wei L [33]	2017	China	Asian	OC	116/110	PCR	HB	7
Catalano [61]	2018	Czech	Caucasian	CRC	1424/1114	TaqMan	HB	7
Pirdelkhosh [62]	2018	Iran	Asian	NSCLC	206/173	PCR-RFLP	PB	8
Zhao YC [34]	2018	China	Asian	CRC	426/500	PCR-RFLP	HB	6
Shamsdin [63]	2018	Iran	Caucasian	CRC	76/73	PCR-RFLP	HB	6
Gabriela V [64]	2018	Brazil	Caucasian	Melanoma	250/250	PCR	PB	8
Fathi F [65]	2018	Iran	Asian	HNSCC	150/150	PCR-RFLP	HB	6
Xie [66]	2018	China	Asian	HCC	225/200	Sequencing	HB	7
Kasamatsu T [67]	2019	Japan	Asian	Myeloma	124/211	PCR-RFLP	PB	7
Fathi F [68]	2019	Iran	Asian	BCC	210/320	PCR-RFLP	HB	7
Ramzi [69]	2020	Iran	Asian	Leukemia	59/38	PCR-RFLP	HB	6
Karami S [70]	2020	Iran	Asian	BC	260/260	PCR-RFLP	HB	6
Demirci [71]	2020	Turkey	Asian	HCC	137/136	TaqMan	HB	7
Wagner W [13]	2020	Poland	Caucasian	RCC	237/260	TaqMan	PB	9
Zang B [72]	2020	China	Asian	EC	814/961	TaqMan	PB	9
Fathi F [73]	2021	Iran	Asian	BCC	210/220	PCR-RFLP	HB	6
Cevik M [74]	2021	Turkey	Caucasian	CRC	103/86	MassArray	HB	6
Al-Harbi [75]	2022	Saudi Arabian	Asian	CRC	100/100	TaqMan	HB	7
Wu [76]	2023	China	Asian	AML	285/342	MassArray	HB	7
Katarzyna [77]	2023	Poland	Caucasian	BC	30/30	TaqMan	HB	6
Hlaing [78]	2023	Japan	Asian	UCC	256/211	PCR-RFLP	HB	7

PB, Population-based; HB, Hospital-based; PCR-RFLP, Polymerase chain reaction-restriction fragment length polymorphism; ARMS-PCR, Amplification refractory mutation system-polymerase chain reaction; HWE, Hardy-Weinberg equilibrium; CC, cervical cancer; BC, breast cancer; HCC, hepatocellular carcinoma; CRC, colorectal cancer; TH, thyroid cancer; EC, esophageal cancer; OC, ovarian cancer; NSCLC, non-small cell lung cancer; RCC, renal cell carcinoma; BCC, basal cell carcinoma; AML, acute myelocytic leukemia.

### Meta-analysis results of PD-1.5 (rs2227981) C/T polymorphism

**Figure 2:**
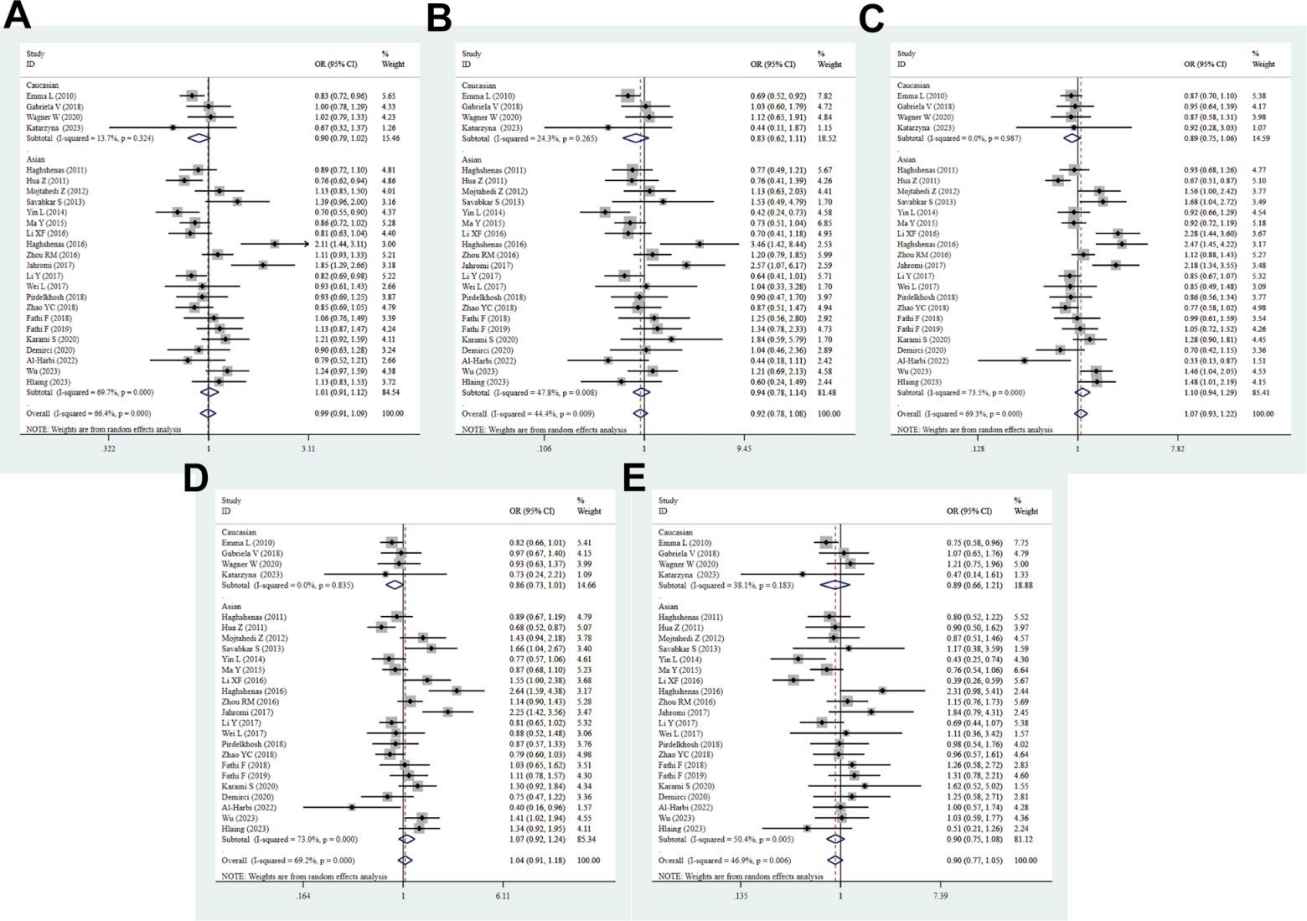
**Forest plot for the association between PD-1.5 gene polymorphism and cancer risk under all the five models.** For each publication, the estimation of OR and its 95% CI was plotted with a box and a horizontal line. The diamonds represented the pooled ORs and 95% CIs. (**A**) Allele model; (**B**) homozygote model; (**C**) heterozygote model; (**D**) dominant model; (**E**) recessive model.

A total of 25 studies with 7724 cases and 7320 controls were included in the meta-analysis to detect the association between PD-1.5 variation and cancer risk. The pooled ORs suggested no significant correlation between the PD-1.5 genotype and cancer susceptibility in all genetic models (T vs. C: OR = 0.99, 95% CI = 0.90-1.09, *P* = 0.807; TT vs. CC: OR = 0.93, 95% CI = 0.79-1.10, *P* = 0.386; CT vs. CC: OR = 1.05, 95% CI = 0.92-1.21, *P* = 0.476; TT+CT vs. CC: OR = 1.03, 95% CI = 0.90-1.17, *P* = 0.694; TT vs. CT+CC: OR = 0.92, 95% CI = 0.78-1.08, *P* = 0.288, Figure 2 and Table 2). Likewise, strong evidence of heterogeneity was found in each comparison, and then we conducted further subgroup analyses to determine the influence of confounding factors. These data showed that PD-1.5 was closely associated with risk of CC (T vs. C: OR = 0.83, 95% CI = 0.73-0.93, *P* = 0.002; TT vs. CC: OR = 0.69, 95% CI = 0.54-0.89, *P* = 0.004), GC (CT vs. CC: OR = 1.68, 95% CI = 1.04-2.72, *P* = 0.036; TT+CT vs. CC: OR = 1.66, 95% CI = 1.04-2.67, *P* = 0.035), NSCLC (T vs. C: OR = 0.83, 95% CI = 0.72-0.95, *P* = 0.009; TT vs. CC: OR = 0.65, 95% CI = 0.44-0.97, *P* = 0.036; TT+CT vs. CC: OR = 0.84, 95% CI = 0.71-0.99, *P* = 0.043), TC (T vs. C: OR = 2.12, 95% CI = 1.44-3.11, *P* = 0.000; TT vs. CC: OR = 3.47, 95% CI = 0.54-0.89, *P* = 0.004; CT vs. CC: OR = 2.48, 95% CI = 1.45-4.22, *P* = 0.001; TT+CT vs. CC: OR = 2.64, 95% CI = 1.59-4.38, *P* = 0.000), Brain tumor (T vs. C: OR = 1.85, 95% CI = 1.29-2.66, *P* = 0.001; TT vs. CC: OR = 2.57, 95% CI = 1.07-6.17, *P* = 0.035; CT vs. CC: OR = 2.19, 95% CI = 1.34-3.55, *P* = 0.001; TT+CT vs. CC: OR = 2.25, 95% CI = 1.42-3.56, *P* = 0.001), OC (T vs. C: OR = 0.84, 95% CI = 0.71-0.99, *P* = 0.036), AML (CT vs. CC: OR = 1.46, 95% CI = 1.04-2.05, *P* = 0.028; TT+CT vs. CC: OR = 1.41, 95% CI = 1.02-1.94, *P* = 0.036) and UCC (CT vs. CC: OR = 1.48, 95% CI = 1.01-2.19, *P* = 0.047). In stratified analysis by ethnicity, there was no remarkable correlation between the rs2227981 polymorphism and cancer risk in all genetic models, and so was it in subgroup analysis by source of controls and quality scores. The rs2227981 mutation was dramatically related to cancer risk in lager sample size (TT vs. CC: OR = 0.78, 95% CI = 0.61-1.00, *P* = 0.048; TT vs. CT+CC: OR = 0.80, 95% CI = 0.66-0.97, *P* = 0.021, Supplementary Table 3). It showed that heterogeneity existed in all genetic models of overall analysis, Asian, higher quality score, and BC. The random effect model was applied to make a reliable result.

**Table 2:** Results of meta-analysis in the PD-1 and PD-L1 gene polymorphisms.

**SNP**	**Model**	**OR (95% CI)**	***P***	***I^2^*(%)**	***P_(H)_***	**Effect model**
PD-1.5 rs2227981C/T	Allelic (T vs. C)	0.99 (0.91, 1.09)	0.879	66.4	0.000	REM
	Homozygous (TT vs. CC)	0.92 (0.78, 1.08)	0.305	44.4	0.009	REM
	Heterozygous (CT vs. CC)	1.07 (0.93, 1.22)	0.350	69.3	0.000	REM
	Dominant (TT+CT vs. CC)	1.04 (0.91, 1.18)	0.568	69.2	0.000	REM
	Recessive (TT vs. CT+CC)	0.90 (0.77, 1.05)	0.167	46.9	0.006	REM
PD-1.9 rs2227982C/T	Allelic (T vs. C)	0.98 (0.89, 1.07)	0.629	56.5	0.002	REM
	Homozygous (TT vs. CC)	0.96 (0.77, 1.19)	0.693	64.6	0.000	REM
	Heterozygous (CT vs. CC)	0.98 (0.86, 1.11)	0.744	47.2	0.017	REM
	Dominant (TT+CT vs. CC)	0.98 (0.86, 1.11)	0.694	52.2	0.006	REM
	Recessive (TT vs. CT+CC)	0.97 (0.82, 1.15)	0.702	60.2	0.00	REM
PD-1.3 rs11568821G/A	Allelic (A vs. G)	0.93 (0.73, 1.19)	0.583	70.3	0.000	REM
	Homozygous (AA vs. GG)	1.33 (0.90, 1.97)	0.156	38.1	0.095	FEM
	Heterozygous (GA vs. GG)	0.83 (0.72, 0.96)	0.012^*^	36.7	0.089	FEM
	Dominant (AA+GA vs. GG)	0.90 (0.71, 1.13)	0.360	58.3	0.004	REM
	Recessive (AA vs. GA+GG)	1.34 (0.92, 1.96)	0.124	20.8	0.246	FEM
PD-1.1 rs36084323G/A	Allelic (A vs. G)	0.93 (0.78,1.10)	0.380	85.7	0.000	REM
	Homozygous (AA vs. GG)	1.14 (0.89, 1.47)	0.300	68.8	0.000	REM
	Heterozygous (GA vs. GG)	0.88 (0.72, 1.09)	0.247	76.4	0.000	REM
	Dominant (AA+GA vs. GG)	0.88 (0.70, 1.11)	0.294	82.9	0.000	REM
	Recessive (AA vs. GA+GG)	1.12 (0.93, 1.34)	0.224	59.6	0.004	REM
PD-1 rs7421861T/C	Allelic (C vs. T)	1.02 (0.89, 1.17)	0.734	73.7	0.000	REM
	Homozygous (CC vs. TT)	0.90 (0.75, 1.08)	0.246	18.3	0.285	FEM
	Heterozygous (CT vs. TT)	1.05 (0.88, 1.24)	0.582	70.3	0.001	REM
	Dominant (TT+CT vs. CC)	1.03 (0.87, 1.22)	0.717	72.3	0.001	REM
	Recessive (CC vs. CT+TT)	0.89 (0.77, 1.03)	0.107	5.6	0.387	FEM
PD-L1 rs4143815G/C	Allelic (C vs. G)	0.85 (0.72, 1.01)	0.063	84.2	0.000	REM
	Homozygous (CC vs. GG)	0.73 (0.52, 1.03)	0.072	82.6	0.000	REM
	Heterozygous (CG vs. GG)	0.78 (0.59, 1.04)	0.091	81.1	0.000	REM
	Dominant (CC+CG vs. GG)	0.76 (0.57, 1.03)	0.074	84.8	0.000	REM
	Recessive (CC vs. CG+GG)	0.86 (0.71, 1.04)	0.123	68.0	0.000	REM
PD-1.6 rs10204525A/G	Allelic (G vs. A)	0.98 (0.86, 1.11)	0.761	75.5	0.000	REM
	Homozygous (GG vs. AA)	0.98 (0.73, 1.32)	0.904	70.1	0.000	REM
	Heterozygous (GA vs. AA)	1.02 (0.88, 1.18)	0.770	61.0	0.002	REM
	Dominant (GG+GA vs. AA)	1.01 (0.86, 1.19)	0.898	70.8	0.000	REM
	Recessive (GG vs. GA+AA)	0.96 (0.79, 1.17)	0.673	57.5	0.005	REM
PD-L1 rs2890658A/C	Allelic (C vs. A)	1.03 (0.75, 1.42)	0.859	85.2	0.000	REM
	Homozygous (CC vs. AA)	0.91 (0.59, 1.41)	0.671	9.4	0.357	FEM
	Heterozygous (CA vs. AA)	1.14 (0.82, 1.57)	0.441	67.4	0.002	REM
	Dominant (CC+CA vs. AA)	1.11 (0.79, 1.56)	0.562	72.9	0.000	REM
	Recessive (CC vs. CA+AA)	0.81 (0.50, 1.32)	0.400	57.3	0.016	REM
PD-L1 rs10815225G/C	Allelic (C vs. G)	1.00 (0.69, 1.43)	0.957	73.9	0.022	REM
	Homozygous (CC vs. GG)	0.84 (0.41, 1.68)	0.613	36.5	0.207	FRM
	Heterozygous (CG vs. GG)	1.03 (0.61,1.77)	0.903	85.3	0.001	REM
	Dominant (CC+CG vs. GG)	1.01 (0.63, 1.63)	0.958	81.9	0.004	REM
	Recessive (CC vs. CG+GG)	0.80 (0.40, 1.62)	0.538	41.7	0.180	FRM
PD-L1 rs17718883C/G	Allelic (G vs. C)	0.07 (0.20, 0.25)	0.000^*^	85.1	0.022	REM
	Homozygous (GG vs. CC)	0.04 (0.01, 0.12)	0.000^*^	0.0	0.829	FEM
	Heterozygous (CG vs. CC)	0.07 (0.02, 0.30)	0.000^*^	84.4	0.002	REM
	Dominant (GG+CG vs. CC)	0.06 (0.02, 0.24)	0.000^*^	84.6	0.001	REM
	Recessive (GG vs. CG+CC)	0.06 (0.02, 0.20)	0.000^*^	0.0	0.855	FEM
PD-L1 rs2297136G/C	Allelic (C vs. G)	1.00 (0.75, 1.35)	0.982	71.4	0.015	REF
	Homozygous (CC vs. GG)	0.85 (0.45, 1.62)	0.624	58.8	0.065	REF
	Heterozygous (CG vs. GG)	1.04 (0.57, 1.90)	0.889	87.3	0.000	REF
	Dominant (CC+CG vs. GG)	1.03 (0.59, 1.41)	0.924	82.8	0.001	REF
	Recessive (CC vs. CG+GG)	0.86 (0.51, 1.44)	0.554	55.7	0.079	REF

*P*, *P*-value of Z-test for statistical significance; P_H_, P-value of Q-test for heterogeneity test. ^*^P<0.05.

### Meta-analysis results of PD-1.9 (rs2227982) C/T polymorphism

**Figure 3:**
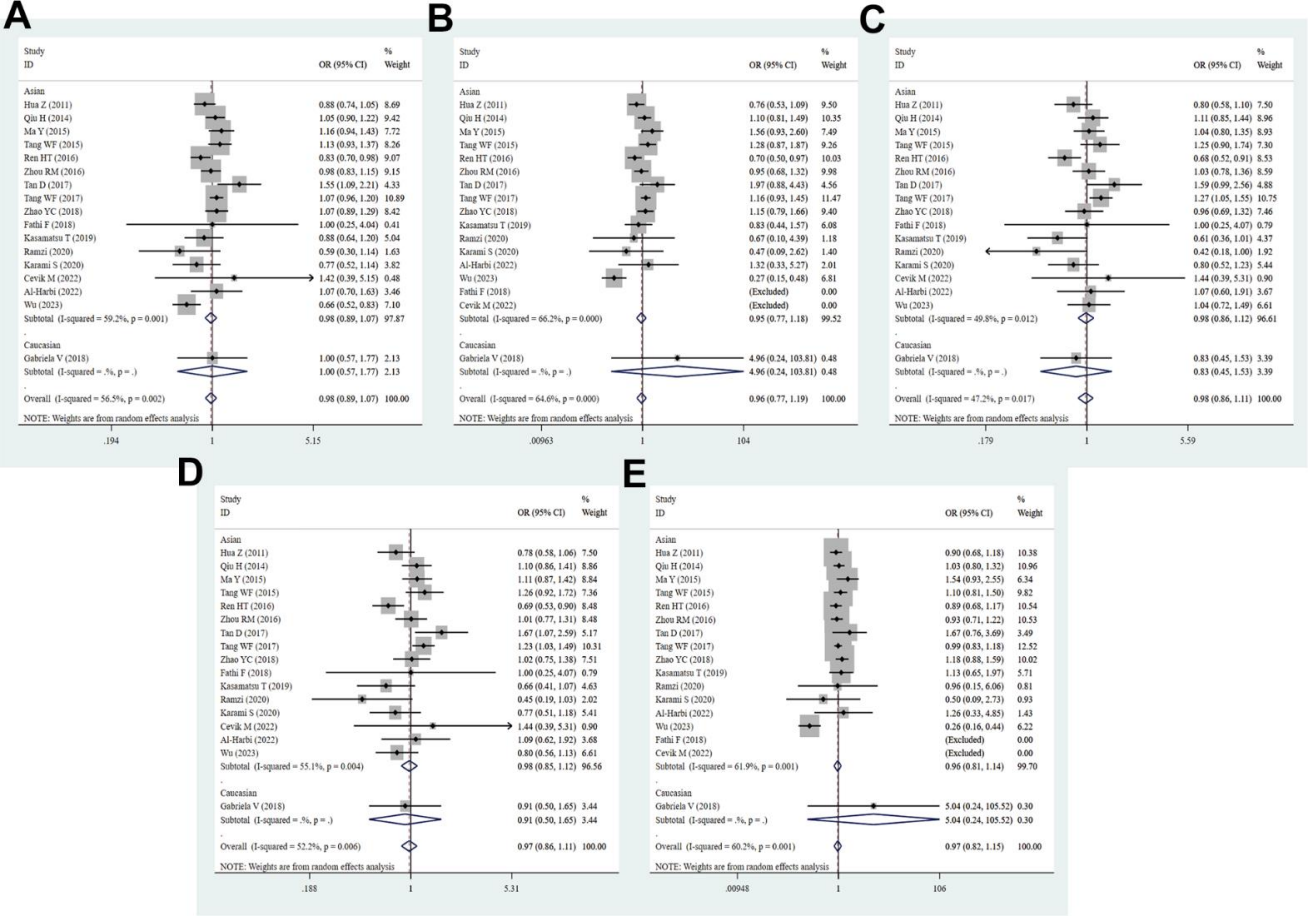
**Forest plot for the association between PD-1.9 gene polymorphism and cancer risk under all the five models.** For each publication, the estimation of OR and its 95% CI was plotted with a box and a horizontal line. The diamonds represented the pooled ORs and 95% CIs. (**A**) Allele model; (**B**) homozygote model; (**C**) heterozygote model; (**D**) dominant model; (**E**) recessive model.

Through the pooled analysis of genetic data of 6029 cases and 7310 controls in a total of 17 studies, of which 2 studies were performed in Caucasians, 15 studies were in Asians. Overall, there was no evident relation between the PD-1.9 and cancer risk (T vs. C: OR = 0.98, 95% CI = 0.89-1.07, *P* = 0.629; TT vs. CC: OR = 0.96, 95% CI = 0.77-1.19, *P* = 0.693; CT vs. CC: OR = 0.98, 95% CI = 0.86-1.11, *P* = 0.744; TT+CT vs. CC: OR = 0.98, 95% CI = 0.86-1.11, *P* = 0.694; TT vs. CT+CC: OR = 0.97, 95% CI = 0.82-1.15, *P* = 0.702, Figure 3 and Table 2). Interestingly, the PD-1.9 T-allele prominently reduced the risk of BC (T vs. C: OR = 0.85, 95% CI = 0.75-0.95, *P* = 0.004; TT vs. CC: OR = 0.72, 95% CI = 0.57-0.92, *P* = 0.007; CT vs. CC: OR = 0.74, 95% CI = 0.61-0.90, *P* = 0.002; TT+CT vs. CC: OR = 0.74, 95% CI = 0.61-0.88, *P* = 0.001) and AML (T vs. C: OR = 0.66, 95% CI = 0.53-0.83, *P* = 0.000; TT vs. CC: OR = 0.27, 95% CI = 0.15-0.48, *P* = 0.000; TT vs. CC+CT: OR = 0.26, 95% CI = 0.16-0.44, *P* = 0.000), whereas the variant was correlated with enhanced risk of EC (CT vs. CC: OR = 1.17, 95% CI = 1.02-1.34, *P* = 0.028; TT+CT vs. CC: OR = 1.14, 95% CI = 1.00-1.30, *P* = 0.047) and OC (T vs. C: OR = 1.55, 95% CI = 1.09-2.21, *P* = 0.016; TT+CT vs. CC: OR = 1.67, 95% CI = 1.07-2.59, *P* = 0.023, Supplementary Table 3). Stratified analyses by ethnicity, source of controls, quality scores and sample size revealed no significant associations with cancer risk in five genetic comparisons. Results of heterogeneity test showed that heterogeneity exists in all the genetic models, so the random effect model was applied to obtain a reliable result.

### Meta-analysis results of PD-1.3 (rs11568821) G/A polymorphism

**Figure 4:**
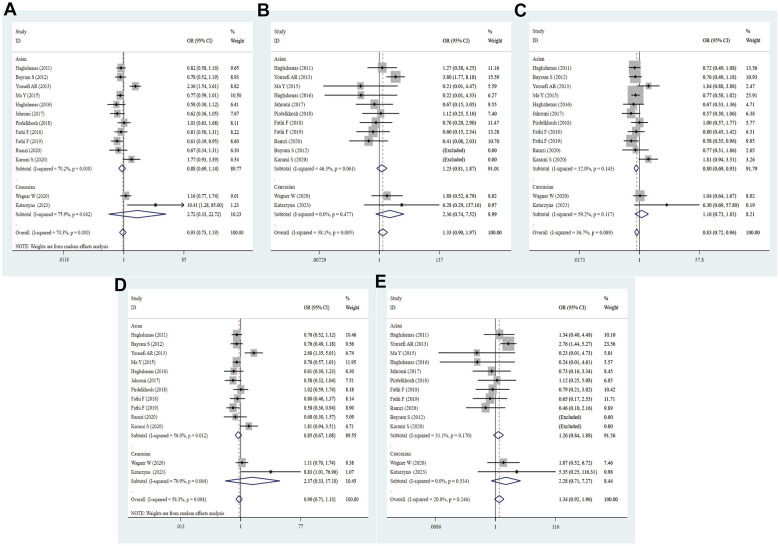
**Forest plot for the association between PD-1.3 gene polymorphism and cancer risk under all the five models.** For each publication, the estimation of OR and its 95% CI was plotted with a box and a horizontal line. The diamonds represented the pooled ORs and 95% CIs. (**A**) Allele model; (**B**) homozygote model; (**C**) heterozygote model; (**D**) dominant model; (**E**) recessive model.

A total of thirteen studies involving 2620 patients and 2733 controls examined the association of PD-1.3 with cancer predisposition. Overall, a notably decreased cancer risk was found in the heterozygous model (GA vs. GG: OR = 0.82, 95% CI = 0.71–0.95, *P* = 0.008, Figure 4 and Table 2). Similarly, the association remained statistically significant in Asian population (GA vs. GG: OR = 0.80, 95% CI = 0.69-0.93, *P* = 0.004), PB (A vs. G: OR = 0.83, 95% CI = 0.68-0.99, *P* = 0.042; GA vs. GG: OR = 0.79, 95% CI = 0.66-0.94, *P* = 0.009; TT+CT vs. CC: OR = 0.80, 95% CI = 0.67-0.95, *P* = 0.012) and high quality score (GA vs. GG: OR = 0.81, 95% CI = 0.67-0.98, *P* = 0.030; AA+GA vs. GG: OR = 0.82, 95% CI = 0.69-0.99, *P* = 0.039). However, the PD-1.3 variant evidently increased the risk of CRC (A vs. G: OR = 2.36, 95% CI = 1.54-3.61, *P* = 0.000; AA vs. GG: OR = 3.80, 95% CI = 1.77-8.18, *P* = 0.001; AA+GA vs. GG: OR = 2.60, 95% CI = 1.35-5.01, *P* = 0.004; AA vs. GG+GA: OR = 2.76, 95% CI = 1.44-5.27, *P* = 0.002). Moreover, the allelic, heterozygous and dominant models of PD-1.3 were remarkably linked with lower risk of BCC (A vs. G: OR = 0.61, 95% CI = 0.39-0.95, *P* = 0.028; GA vs. GG: OR = 0.58, 95% CI = 0.35-0.96, *P* = 0.035; AA+GA vs. GG: OR = 0.58, 95% CI = 0.36-0.94, *P* = 0.028, Supplementary Table 3). Subgroup analysis based on sample size manifested no remarkable association between PD-1.3 polymorphism and cancer risk in any genetic models. It showed that heterogeneity existed in the allelic and domain models of overall group and Asian, but no heterogeneity was found in NSCLC subgroup.

### Meta-analysis results of PD-1.1 (rs36084323) G/A polymorphism

**Figure 5:**
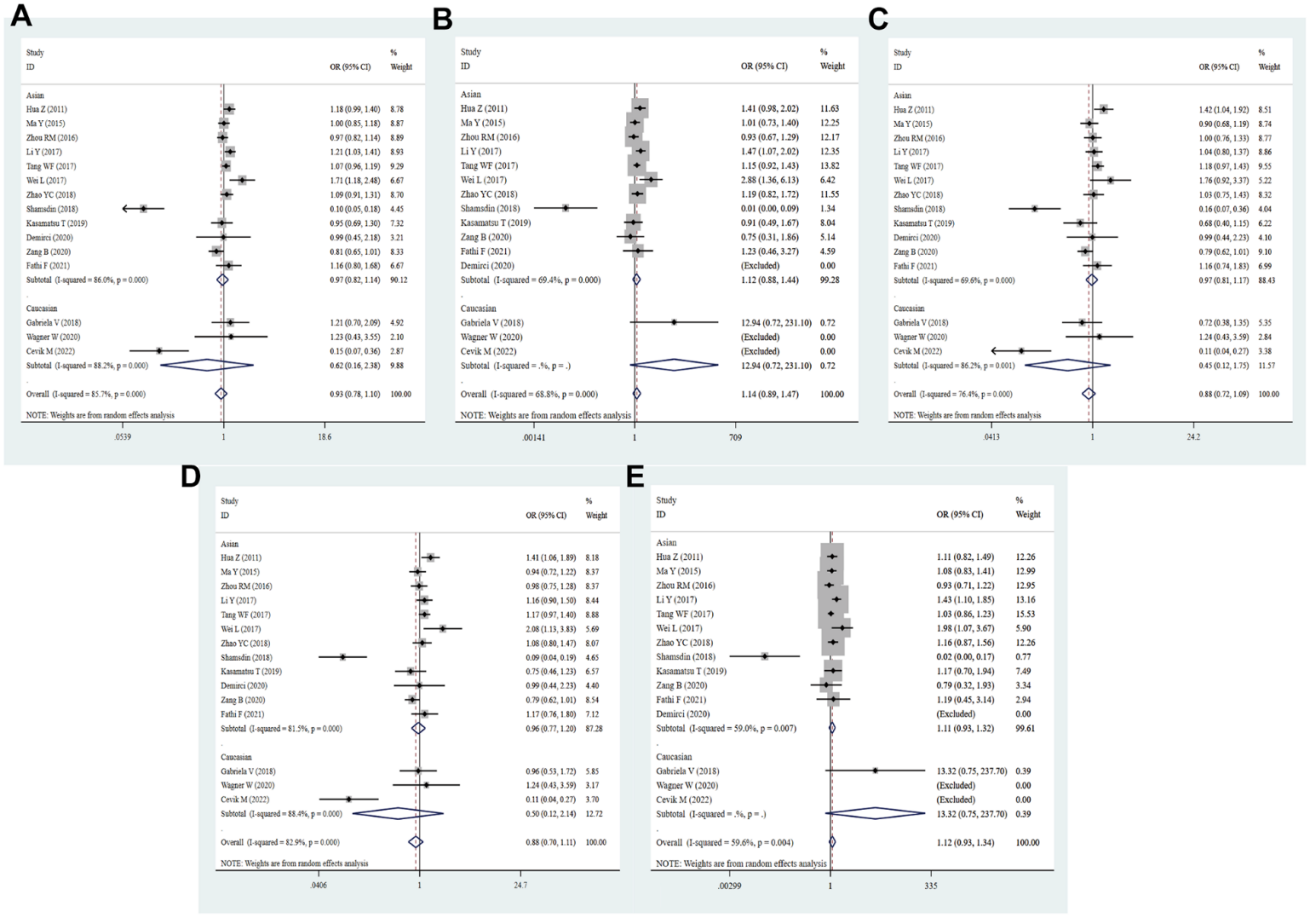
**Forest plot for the association between PD-1.1 gene polymorphism and cancer risk under all the five models.** For each publication, the estimation of OR and its 95% CI was plotted with a box and a horizontal line. The diamonds represented the pooled ORs and 95% CIs. (**A**) Allele model; (**B**) homozygote model; (**C**) heterozygote model; (**D**) dominant model; (**E**) recessive model.

Fifteen studies with 5693 cases and 6749 controls were qualified for the association between PD-1.1 SNP and cancer predisposition. Among these eligible studies, four studies were from the Caucasians, and eleven studies from Asians. The pooled analysis disclosed no relevance between PD-1.1 variation and cancer risk (A vs. G: OR = 0.93, 95% CI = 0.78-1.10, *P* = 0.380; AA vs. GG: OR = 1.14, 95% CI = 0.89-1.47, *P* = 0.300; GA vs. GG: OR = 0.88, 95% CI = 0.72-1.09, *P* = 0.247; AA+GA vs. GG: OR = 0.88, 95% CI = 0.70-1.11, *P* = 0.294; AA vs. GA+GG: OR = 1.12, 95% CI = 0.93-1.34, *P* = 0.224, Figure 5 and Table 2). The carriers with PD-1.1 A-allele was slightly related to increased risk.of BC (GA vs. GG: OR = 1.42, 95% CI = 1.04-1.93, *P* = 0.026; AA+GA vs. GG: OR = 1.41, 95% CI = 1.06-1.89, *P* = 0.020) and OC (AA vs. GA+GG: OR = 1.50, 95% CI = 1.18-1.91 *P* = 0.185). There was a positive relevance between the PD-1.1 variant and cancer predisposition in the Asian descents (AA vs. GG: OR = 1.18, 95% CI = 1.02-1.38, *P* = 0.032; AA vs. GA+GG: OR = 1.12, 95% CI = 1.01-1.25, *P* = 0.039, Supplementary Table 3), indicating that the PD-1.1varant might serve as a risk factor in Asians. Not only that, further stratification analyses by type of cancer, source of control, quality score and sample size also revealed similar results. Heterogeneity was found to be present in all genetic models, so a random effect pattern was selected.

### Meta-analysis results of PD-1 rs7421861 T/C polymorphism

**Figure 6:**
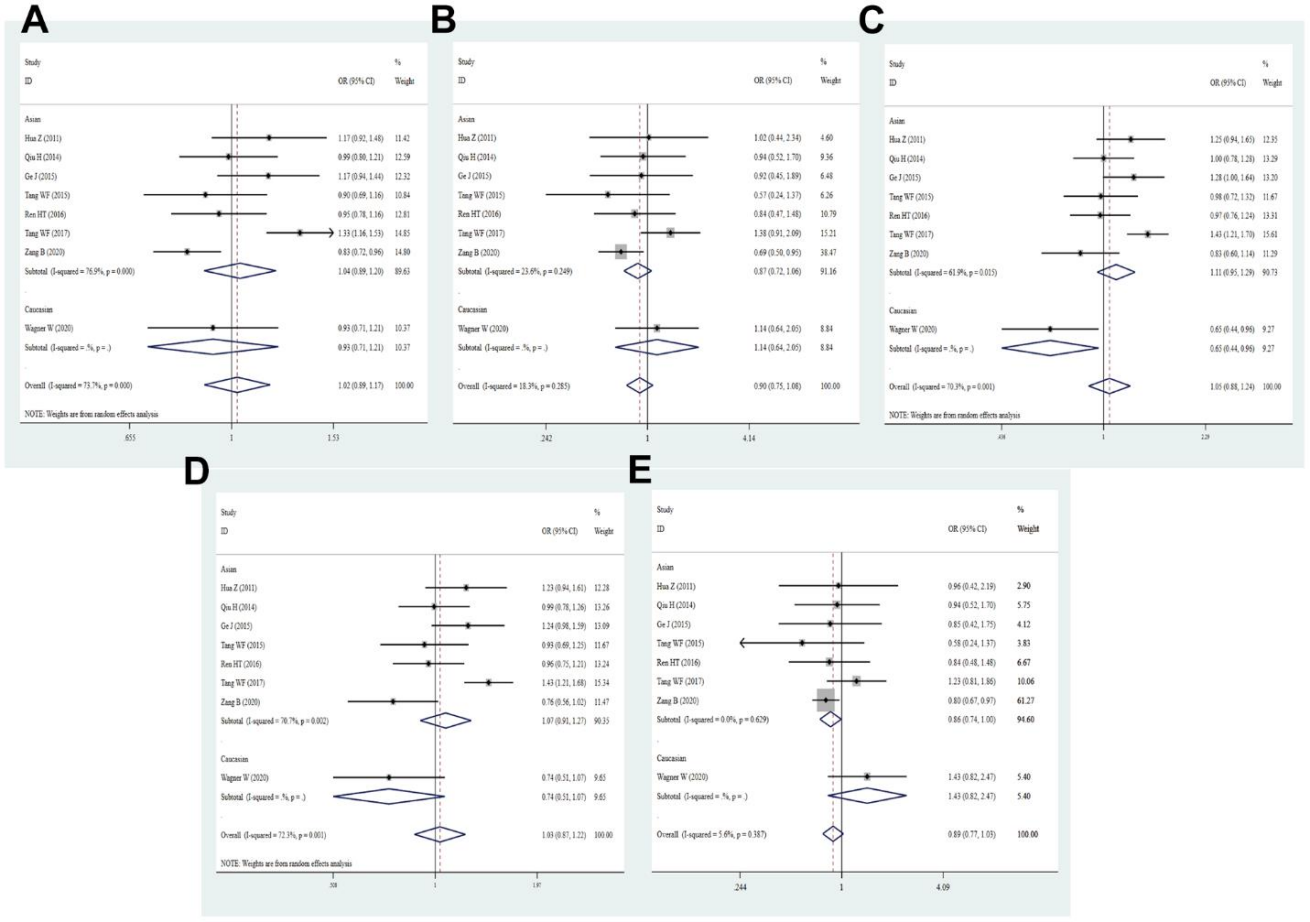
**Forest plot for the association between rs7421861 gene polymorphism and cancer susceptibility under all the five models.** For each publication, the estimation of OR and its 95% CI was plotted with a box and a horizontal line. The diamonds represented the pooled ORs and 95% CIs. (**A**) Allele model; (**B**) homozygote model; (**C**) heterozygote model; (**D**) dominant model; (**E**) recessive model.

Eight eligible studies with 4632 patients and 5873 controls reported association of PD-1 rs7421861 polymorphism with cancer risk in our study. In overall analysis, no significant association with cancer susceptibility was found (C vs. T: OR = 1.02, 95% CI = 0.89-1.17, *P* = 0.734; CC vs. TT: OR = 0.90, 95% CI = 0.75-1.24, *P* = 0.246; CT vs. TT: OR = 1.05, 95% CI = 0.88-1.24, *P* = 0.582; TT+CT vs. TT: OR = 1.07, 95% CI = 0.91-1.27, *P* = 0.717; CC vs. CT+TT: OR = 0.89, 95% CI = 0.77-1.03, *P* = 0.107, Figure 6 and Table 2). And then, we did not detect the relationship between the rs7421861 variation and cancer risk in subgroups of ethnicity, source of control, quality score and sample size. The heterozygote and dominant models of rs7421861 were significantly correlated with enhanced risk of BC (CT vs. TT: OR = 1.42, 95% CI = 1.04-1.93, *P* = 0.026; TT+CT vs. TT: OR = 1.41, 95% CI = 1.06-1.89, *P* = 0.020) and OC (CC vs. CT+TT: OR = 1.50, 95% CI = 1.18-1.91, *P* = 0.001 Supplementary Table 3). As heterogeneity of the rs7421861 existed in allele, heterozygote and dominant models, the random effects model was selected in the above models.

### Meta-analysis results of PD-L1 rs4143815 G/C polymorphism

**Figure 7:**
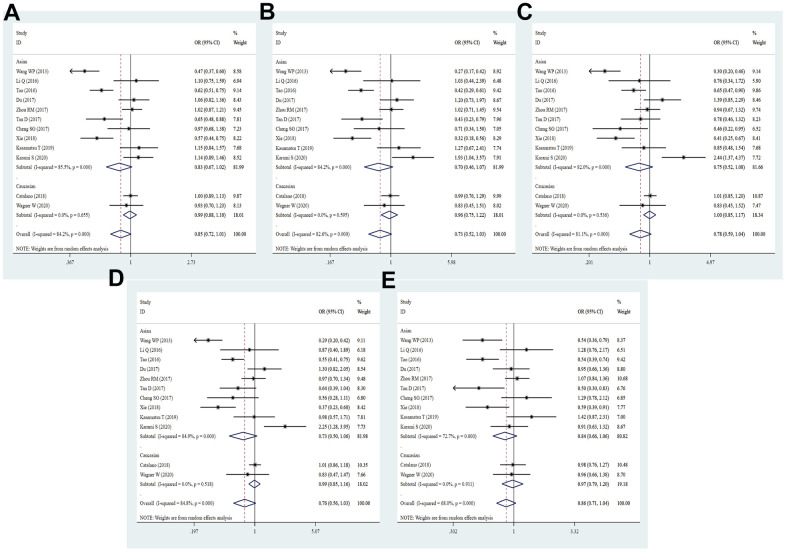
**Forest plot for the association between PD-L1 rs4143815 gene polymorphism and cancer susceptibility under all the five models.** For each publication, the estimation of OR and its 95% CI was plotted with a box and a horizontal line. The diamonds represented the pooled ORs and 95% CIs. (**A**) Allele model; (**B**) homozygote model; (**C**) heterozygote model; (**D**) dominant model; (**E**) recessive model.

A total of 12 eligible studies embodying 4008 cases and 4147 controls were examined for correlation of PD-L1 rs4143815 with the risk of cancer. Overall, there was no statistically significant association between the rs4143815 SNP and cancer risk in all genetic models (C vs. G: OR = 0.85, 95% CI = 0.72-1.01, *P* = 0.063; CC vs. GG: OR = 0.73, 95% CI = 0.52-1.03, *P* = 0.072; CG vs. GG: OR = 0.78, 95% CI = 0.59-1.04, *P* = 0.091; CC+CG vs. GG: OR = 0.76, 95% CI = 0.57-1.03, *P* = 0.074; CC vs. CG+GG: OR = 0.86, 95% CI = 0.71-1.04, *P* = 0.123, Figure 7 and Table 2). When stratified analysis was performed by ethnicity, we identified no significant relevance. According to subgroup analyses by quality score, source of control and sample size, there was no dramatic relationship between the rs4143815 polymorphism and cancer risk. Intriguingly, the rs4143815 mutation was markedly associated with risk of GC (C vs. G: OR = 0.66, 95% CI = 0.45-0.99, *P* = 0.045; CC vs. GG: OR = 0.44, 95% CI = 0.24-0.81, *P* = 0.008; CG vs. GG: OR = 0.51, 95% CI = 0.29-0.92, *P* = 0.025; CC+CG vs. GG: OR = 0.49, 95% CI = 0.28- 0.85, *P* = 0.012), OC (C vs. G: OR = 0.65, 95% CI = 0.48-0.88, *P* = 0.005; CC vs. GG: OR = 0.43, 95% CI = 0.23-0.79, *P* = 0.006; CC vs. GG+CG: OR = 0.50, 95% CI = 0.30-0.83, *P* = 0.007), HCC (CC vs. GG: OR = 0.46, 95% CI = 0.21-0.99, *P* = 0.047; CG vs. GG: OR = 0.42, 95% CI = 0.28-0.64, *P* = 0.000; CC+CG vs. GG: OR = 0.43, 95% CI = 0.29-0.63, *P* = 0.000) and BC (CC vs. GG: OR = 1.93, 95% CI = 1.04-3.57, *P* = 0.037; CG vs. GG: OR = 2.45, 95% CI = 1.37-4.37, *P* = 0.003; CC+CG vs. GG: OR = 2.25, 95% CI = 1.28-3.95, *P* = 0.005, Supplementary Table 3). These data with high heterogeneity applied the random-effect model for quantitative synthesis.

### Meta-analysis results of PD-1.6 (rs10204525) A/G polymorphism

**Figure 8:**
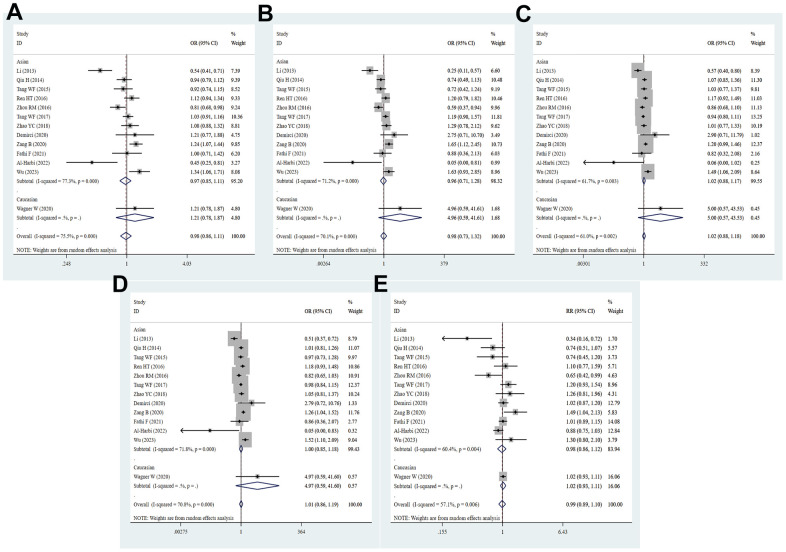
**Forest plot for the association between PD-1.6 gene polymorphism and cancer susceptibility under all the five models.** For each publication, the estimation of OR and its 95% CI was plotted with a box and a horizontal line. The diamonds represented the pooled ORs and 95% CIs. (**A**) Allele model; (**B**) homozygote model; (**C**) heterozygote model; (**D**) dominant model; (**E**) recessive model.

By integrating quantitatively allele or genotype distribution of 5528 patients and 6875 controls, we did not discover any significant relationship between the PD-1.6 SNP and cancer risk in five genetic comparisons (G vs. A: OR = 0.98, 95% CI = 0.86-1.11, *P* = 0.761; GG vs. AA: OR = 0.98, 95% CI = 0.73-1.32, *P* = 0.904; GA vs. AA: OR = 1.02, 95% CI = 0.88-1.18, *P* = 0.861; GG+GA vs. AA: OR = 1.01, 95% CI = 0.86-1.19, *P* = 0.898; GG vs. GA+AA: OR = 0.96, 95% CI = 0.79-1.17, *P* = 0.673, Figure 8 and Table 2). As shown in Supplementary Table 3, the PD-1.6 variant was not dramatically associated with cancer susceptibility in stratified analyses by ethnicity, source of control, quality score and sample size. Based on subgroup analysis by type of cancer, the PD-1.6 polymorphism remarkably elevated the risk of AML (G vs. A: OR = 1.34, 95% CI = 1.06-1.71, *P* = 0.017; GA vs. AA: OR = 1.49, 95% CI = 1.06-2.09, *P* = 0.020; GG+GA vs. AA: OR = 1.52, 95% CI = 1.10-2.09, *P* = 0.011, Supplementary Table 3). Heterogeneity results indicated that heterogeneity nearly existed in all genetic models.

### Meta-analysis results of PD-L1 rs2890658 A/C, rs10815225 G/C, rs17718883 C/G, and rs2297136 A/G polymorphisms

Relationships of PD-L1 rs2890658, rs10815225, rs17718883, and rs2297136 with cancer risk were examined in 9 studies, 3 studies, 3 studies, and 4 studies, respectively. All studies about these four SNPs are conducted in Asian population (Supplementary Figure 1). In general, an obvious association between PD-L1 rs17718883 SNP and lower cancer risk was discovered in all genetic models (G vs. C: OR = 0.07, 95% CI = 0.20-0.25, P = 0.000; GG vs. CC: OR = 0.04, 95% CI = 0.01-0.12, P = 0.000; GC vs. CC: OR = 0.07, 95% CI = 0.02-0.30, P = 0.000; GG+GC vs. CC: OR = 0.06, 95% CI = 0.02-0.24, P = 0.000; GG vs. GC+CC: OR = 0.06, 95% CI = 0.02-0.20, P = 0.000, Table 2). Similar results were detected in subgroups of Asian, PB, small sample size, low quality score (G vs. C: OR = 0.12, 95% CI = 0.04-0.35, P = 0.000; GG vs. CC: OR = 0.05, 95% CI = 0.01-0.16, P = 0.000; GC vs. CC: OR = 0.12, 95% CI = 0.03-0.48, *P* = 0.000; GG+GC vs. CC: OR = 0.10, 95% CI = 0.03-0.36, *P* = 0.000; GG vs. GC+CC: OR = 0.07, 95% CI = 0.02-0.24, *P* = 0.000) and high quality score (G vs. C: OR = 0.01, 95% CI = 0.00-0.08, *P* = 0.000; GG vs. CC: OR = 0.02, 95% CI = 0.00-0.32, *P* = 0.006). However, we did not detect any associations between other three SNPs and risk of cancer in subgroup analyses by ethnicity, sample size, source of control and quality scores.

Results demonstrated that PD-L1 rs2890658 was dramatically correlated with the lower risk of HCC (C vs. A: OR = 0.74, 95% CI = 0.55-1.00, *P* = 0.046) and BC (C vs. A: OR = 0.53, 95% CI = 0.40-0.71, *P* = 0.000; CC vs. AA+CA: OR = 0.40, 95% CI = 0.28-0.57, *P* = 0.000), while the variant notably enhanced the risk of NSCLC (C vs. A: OR = 1.72, 95% CI = 1.39-2.13, *P* = 0.000; CA vs. AA: OR = 1.74, 95% CI = 1.37-2.19, *P* = 0.000). The rs10815225 variant significantly decreased the risk of GC (C vs. G: OR = 0.65, 95% CI = 0.45-0.96, *P* = 0.028; GC vs. GG: OR = 0.57, 95% CI = 0.38-0.85, *P* = 0.006; CC+GC vs. GG: OR = 0.60, 95% CI = 0.40-0.89, *P* = 0.0011). Furthermore, the rs17718883 was remarkably correlative with reduced HCC (G vs. C: OR = 0.03, 95% CI = 0.01-0.23, *P* = 0.001; GG vs. CC: OR = 0.03, 95% CI = 0.01-1.13, *P* = 0.000; GC vs. CC: OR = 0.04, 95% CI = 0.01-0.15, *P* = 0.000; GG+GC vs. CC: OR = 0.03, 95% CI = 0.01-0.16, *P* = 0.000; GG vs. CC+GC: OR = 0.05, 95% CI = 0.02-0.20, *P* = 0.000) and GC risk (G vs. C: OR = 0.20, 95% CI = 0.11-0.36, *P* = 0.000; GG vs. CC: OR = 0.06, 95% CI = 0.01-0.43, *P* = 0.005; GC vs. CC: OR = 0.24, 95% CI = 0.12-0.48, *P* = 0.000; GG+GC vs. CC: OR = 0.19, 95% CI = 0.10-0.37, *P* = 0.000; GG vs. CC+GC: OR = 0.08, 95% CI = 0.01-0.60, *P* = 0.014). As for PD-L1 rs2297136, the mutant was closely related to NSCLC (C vs. G: OR = 1.30, 95% CI = 1.00-1.70, *P* = 0.048; GC vs. GG: OR = 2.29, 95% CI = 1.56-3.36, *P* = 0.000; CC+GC vs. GG: OR = 2.09, 95% CI = 1.43-3.04, *P* = 0.000; CC vs. GG+GC: OR = 0.44, 95% CI = 0.20-0.97, *P* = 0.042) and HCC risk (C vs. G: OR = 0.68, 95% CI = 0.49-0.93, *P* = 0.017; CC vs. GG: OR = 0.39, 95% CI = 0.18-0.85, *P* = 0.018; GC vs. GG: OR = 0.24, 95% CI = 0.12-0.48, *P* = 0.000; CC+GC vs. GG: OR = 0.19, 95% CI = 0.10-0.37, *P* = 0.000; CC vs. GG+GC: OR = 0.08, 95% CI = 0.01-0.60, *P* = 0.014, Supplementary Table 3).

### Sensitivity analyses and publication bias

**Figure 9:**
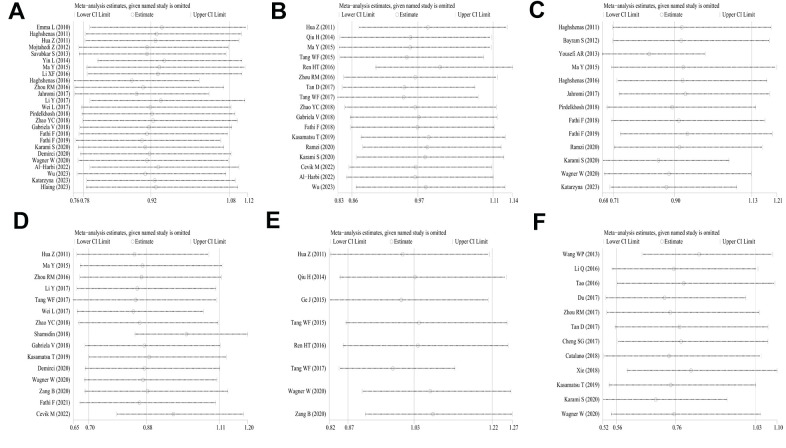
**Analyses of the influence of a single study on the total combined OR in the dominant model.** (**A**) PD-1.5 polymorphism; (**B**) PD-1.9 polymorphism; (**C**) PD-1.3 polymorphism; (**D**) PD-1.1 polymorphism; (**E**) PD-1 rs7421861 polymorphism; (**F**) PD-L1 rs4143815 polymorphism.

Sensitivity analysis was applied to detect individual study’s influence on the composite results by sequentially removing single eligible study and no significant change was observed in certain models, suggesting the credibility of our research results (Figure 9 and Supplementary Figure 2). The Begg’s funnel plot and Egger’s test were applied to assess the potential publication bias in this meta-analysis. Except for the PD-1.5, most of the funnel plots were symmetrical distribution, indicating absence of publication bias (Table 3, Figure 10 and Supplementary Figure 3).

**Table 3:** Publication bias of various models for PD1 and PD-L1 gene polymorphisms.

**Variables**	**Allelic**		**Homozygous**		**Heterozygous**		**Dominant**		**Recessive**	
	***P _B_***	***P_E_***	***P _B_***	***P_E_***	***P _B_***	***P_E_***	***P _B_***	***P_E_***	***P _B_***	***P_E_***
PD-1.5 rs2227981C/T	0.011^*^	0.015^*^	0.071	0.022^*^	0.032^*^	0.033^*^	0.032^*^	0.029	0.091	0.014^*^
PD-1.9 rs2227982C/T	0.893	0.777	0.827	0.810	0.685	0.670	0.620	0.655	0.743	0.843
PD-1.3 rs11568821G/A	0.945	0.852	0.592	0.017^*^	0.631	0.400	0.837	0.436	0.592	0.005^*^
PD-1.1 rs36084323G/A	0.198	0.148	0.631	0.330	0.428	0.189	0.428	0.184	0.631	0.391
PD-1 rs7421861T/C	0.902	0.739	0.711	0.492	0.174	0.018^*^	0.108	0.018^*^	0.711	0.119
PD-L1 rs4143815G/C	0.732	0.563	1.000	0.879	0.945	0.568	1.000	0.607	0.537	0.502
PD-1.6 rs10204525A/G A/Grs10204525A/G	0.373	0.253	0.244	0.322	0.244	0.496	0.304	0.496	0.064	0.059
PD-L1 rs2890658A/C	0.711	0.611	1.000	0.609	0.902	0.309	0.902	0.330	0.902	0.443
PD-L1 rs10815225G/C	1.000	0.679	0.296	0.615	1.000	0.691	1.000	0.690	0.296	0.614
PD-L1 rs17718883C/G	0.296	0.490	1.000	0.687	0.296	0.377	0.296	0.447	1.000	0.687
PD-L1 rs2297136G/C	0.308	0.268	1.000	0.754	0.308	0.210	0.734	0.218	0.734	0.279

*P _B_*, P-value of Begg’s rank correlation test; *P_E_*, P-value of Egger’s linear regression test. ^*^P<0.05.

**Figure 10:**
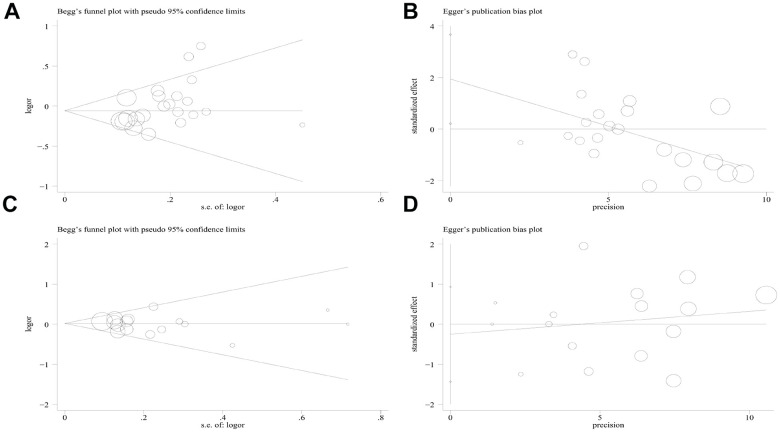
**Begg’s funnel plot and Egger’s linear regression test for the publication biases under the dominant model.** (**A**) Begg’s test for PD-1.5 polymorphism; (**B**) Egger’s test for PD-1.5 polymorphism; (**C**) Begg’s test for PD-1.9 polymorphism; (**D**) Egger’s test for PD-1.9 polymorphism.

**Figure 10:**
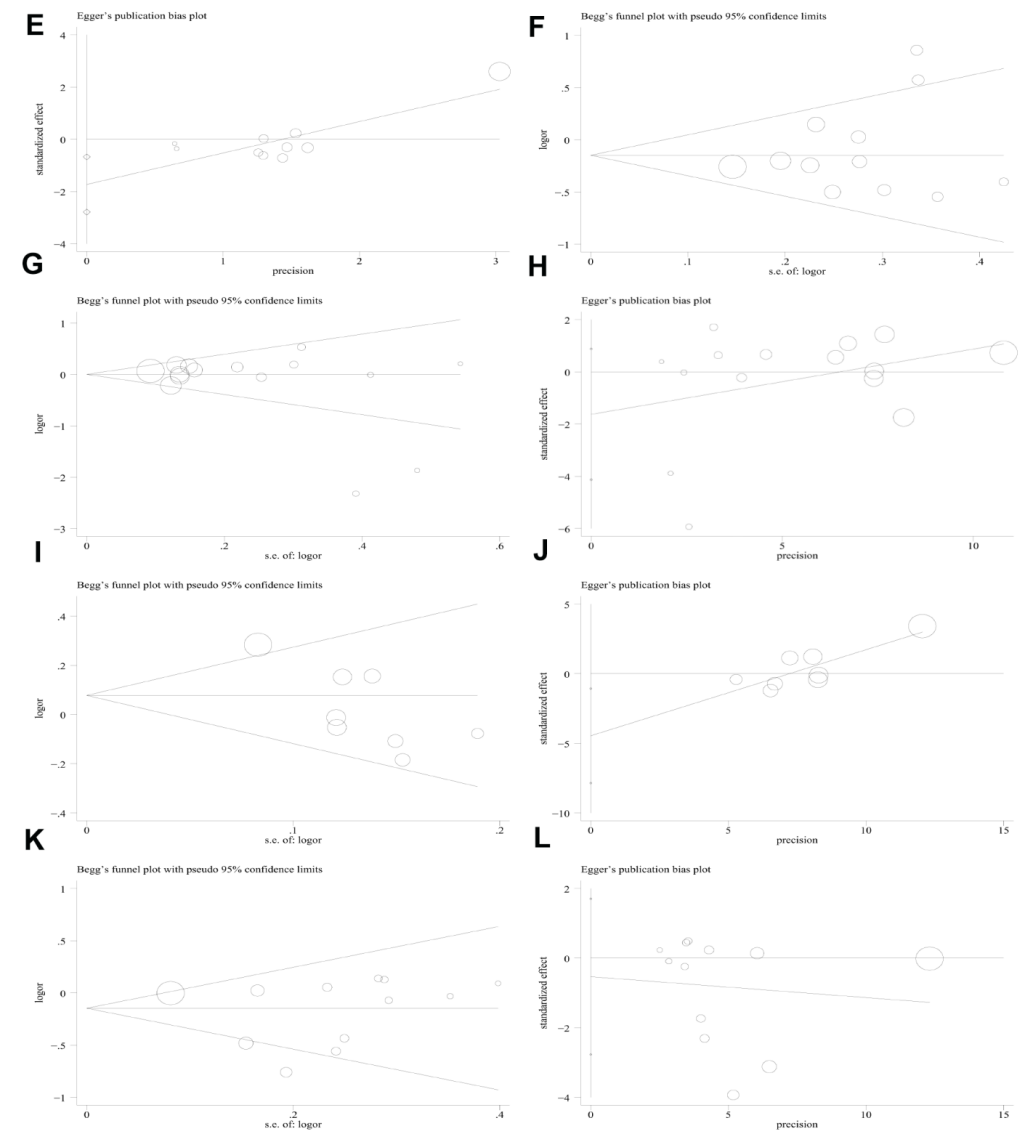
**Begg’s funnel plot and Egger’s linear regression test for the publication biases under the dominant model.** (**E**) Begg’s test for PD-1.3 polymorphism; (**F**) Egger’s test for PD-1.3 polymorphism; (**G**) Begg’s test for PD-1.1 polymorphism; (**H**) Egger’s test for PD-1.1 polymorphism; (**I**) Begg’s test for PD-1 rs7421861 polymorphism; (**J**) Egger’s test for PD-1 rs7421861 polymorphism; (**K**) Begg’s test for PD-L1 rs4143815 polymorphism; (**L**) Egger’s test for PD-L1 rs4143815 polymorphism.

### FPRP results

We investigated determinants of FPRP across a range of probabilities to determine whether a given relationship of PD-1 and PD-L1 SNPs and cancer predisposition is deserving of attention or is noteworthy. Table 4 represents the calculated FPRP values for the main evident findings in this meta-analysis. With the assumption of a prior probability of 0.25, the FPRP values were less than 0.2, implying the observed correlations were significant. The heterozygote model of PD-1.3 was related to cancer risk, as well as the allele model of PD-L1 rs4143815. Similarly, with a prior probability of 0.25, the allele, homozygote, dominant and recessive models of PD-L1rs17718883 was notably associated with cancer risk (*P* < 0.2, Table 4).

**Table 4:** False-positive report probability analysis of the noteworthy results.

**SNP**	**Genetic model**	**OR (95% CI)**	**P**	**Power**	**Prior probability**				
					**0.25**	**0.1**	**0.01**	**0.001**	**0.0001**
PD-1.5 rs2227981C/T	Allele	0.99 (0.91, 1.09)	0.838	1.000	0.715	0.883	0.988	0.999	1.000
	Homozygote	0.92 (0.78, 1.08)	0.308	1.000	0.480	0.735	0.968	0.997	1.000
	Heterozygote	1.07 (0.93, 1.22)	0.312	1.000	0.484	0.737	0.969	0.997	1.000
	Dominant	1.04 (0.91, 1.18)	0.543	1.000	0.620	0.830	0.982	0.998	1.000
	Recessive	0.90 (0.77, 1.05)	0.308	1.000	0.480	0.735	0.968	0.997	1.000
PD-1.9 rs2227982C/T	Allele	0.98 (0.89, 1.07)	0.652	1.000	0.662	0.854	0.985	0.998	1.000
	Homozygote	0.96 (0.77, 1.19)	0.709	1.000	0.680	0.865	0.986	0.999	1.000
	Heterozygote	0.98 (0.86, 1.11)	0.756	1.000	0.694	0.872	0.987	0.999	1.000
	Dominant	0.98 (0.86, 1.11)	0.756	1.000	0.694	0.872	0.987	0.999	1.000
	Recessive	0.97 (0.82, 1.15)	0.726	1.000	0.685	0.867	0.986	0.999	1.000
PD-1.3 rs11568821G/A	Allele	0.93 (0.73, 1.19)	0.564	1.000	0.629	0.835	0.982	0.998	1.000
	Homozygote	1.33 (0.90, 1.97)	0.155	0.979	0.322	0.587	0.940	0.994	0.999
	Heterozygote	0.83 (0.72, 0.96)	0.012	1.000	0.035^*^	0.098^*^	0.545	0.923	0.992
	Dominant	0.90 (0.71, 1.13)	0.364	1.000	0.522	0.766	0.973	0.997	0.997
	Recessive	1.34 (0.92, 1.96)	0.131	0.980	0.287	0.547	0.930	0.993	0.999
PD-1.1 rs36084323G/A	Allele	0.93 (0.78, 1.10)	0.397	1.000	0.543	0.781	0.975	0.997	1.000
	Homozygote	1.14 (0.89, 1.47)	0.312	1.000	0.484	0.738	0.969	0.997	1.000
	Heterozygote	0.88 (0.72, 1.09)	0.242	1.000	0.420	0.685	0.982	0.998	1.000
	Dominant	0.88 (0.70, 1.11)	0.281	1.000	0.457	0.716	0.965	0.996	1.000
	Recessive	1.12 (0.93, 1.34)	0.216	1.000	0.393	0.660	0.955	0.995	1.000
PD-1 rs7421861T/C	Allele	1.02 (0.89, 1.17)	0.778	1.000	0.480	0.735	0.968	0.997	1.000
	Homozygote	0.90 (0.75, 1.08)	0.250	0.996	0.430	0.693	0.961	0.996	1.000
	Heterozygote	1.05 (0.88, 1.24)	0.565	1.000	0.629	0.836	0.982	0.998	1.000
	Dominant	1.07 (0.91, 1.27)	0.439	1.000	0.568	0.798	0.978	0.998	1.000
	Recessive	0.89 (0.77, 1.03)	0.118	1.000	0.261	0.515	0.921	0.992	0.999
PD-L1 rs4143815G/C	Allele	0.85 (0.72, 1.01)	0.065	1.000	0.163^*^	0.368	0.865	0.985	0.998
	Homozygote	0.73 (0.52, 1.03)	0.358	1.000	0.518	0.763	0.973	0.997	1.000
	Heterozygote	0.78 (0.59, 1.04)	0.217	1.000	0.395	0.662	0.956	0.995	1.000
	Dominant	0.76 (0.57, 1.03)	0.190	1.000	0.363	0.631	0.950	0.995	0.999
	Recessive	0.86 (0.71, 1.04)	0.880	1.000	0.706	0.878	0.988	0.999	1.000
PD-1.6 rs10204525A/G	Allele	0.98 (0.86, 1.11)	0.751	1.000	0.692	0.871	0.987	0.999	1.000
	Homozygote	0.98 (0.73, 1.32)	0.894	1.000	0.728	0.889	0.989	0.999	1.000
	Heterozygote	1.02 (0.88, 1.18)	0.790	1.000	0.703	0.877	0.987	0.999	1.000
	Dominant	1.01 (0.86, 1.19)	0.905	1.000	0.731	0.891	0.989	0.999	1.000
	Recessive	0.96 (0.79, 1.17)	0.686	1.000	0.673	0.861	0.985	0.999	1.000
PD-L1 rs2890658A/C	Allele	1.03 (0.75, 1.42)	0.857	1.000	0.720	0.885	0.988	0.999	1.000
	Homozygote	0.91 (0.59, 1.41)	0.673	0.996	0.670	0.859	0.985	0.999	1.000
	Heterozygote	1.14 (0.82, 1.57)	0.422	1.000	0.559	0.492	0.977	0.998	1.000
	Dominant	1.11 (0.79, 1.56)	0.548	1.000	0.622	0.831	0.982	0.998	1.000
	Recessive	0.81 (0.50, 1.32)	0.398	0.974	0.551	0.786	0.976	0.998	1.000
PD-L1 rs10815225G/C	Allele	1.00 (0.69, 1.43)	0.957	1.000	0.742	0.896	0.990	0.999	1.000
	Homozygote	0.84 (0.41, 1.68)	0.622	0.929	0.668	0.858	0.985	0.999	1.000
	Heterozygote	1.03 (0.61,1.77)	0.914	0.992	0.735	0.892	0.989	0.999	1.000
	Dominant	1.01 (0.63, 1.63)	0.967	0.997	0.744	0.897	0.990	0.999	1.000
	Recessive	0.80 (0.40, 1.62)	0.553	0.904	0.640	0.842	0.983	0.998	1.000
PD-L1 rs17718883C/G	Allele	0.07 (0.02, 0.25)	0.000	0.001	0.093^*^	0.236	0.772	0.972	0.997
	Homozygote	0.04 (0.01, 0.12)	0.000	0.000	0.008^*^	0.025^*^	0.218	0.738	0.966
	Heterozygote	0.07 (0.02, 0.30)	0.001	0.000	0.202	0.432	0.893	0.988	0.999
	Dominant	0.06 (0.02, 0.24)	0.000	0.000	0.133^*^	0.315	0.835	0.981	0.998
	Recessive	0.06 (0.02, 0.20)	0.000	0.000	0.048^*^	0.131^*^	0.623	0.943	0.994
PD-L1 rs2297136G/C	Allele	1.00 (0.75, 1.35)	0.949	1.000	0.740	0.895	0.989	0.999	1.000
	Homozygote	0.85 (0.45, 1.62)	0.621	0.947	0.663	0.855	0.985	0.998	1.000
	Heterozygote	1.04 (0.57, 1.90)	0.898	0.983	0.733	0.892	0.989	0.999	1.000
	Dominant	1.03 (0.59, 1.41)	0.854	1.000	0.719	0.885	0.988	0.999	1.000
	Recessive	0.86 (0.51, 1.44)	0.566	0.980	0.634	0.839	0.983	0.998	1.000

^*^P < 0.2.

## DISCUSSION

To our knowledge, environmental factors and genetic susceptibility of an individual exert a pivotal role in the development of tumorigenesis [79, 80]. SNPs can be recognized as the common biological markers, which helps scientists to identify genes associated with complex diseases such as cancer [81]. PD-1 has been identified as powerful candidate genes implicated in the immunosuppressive and antitumor effects [82]. PD-1 negatively regulates the immune response of T-lymphocytes, and binding of PD-1 to its ligands PD-L1 extensively restrains host anti-tumor immunity, creating an anti-tumor suppressive milieu [83, 84]. Over-expression of PD-1 has been reported to facilitate immune system avoidance in different cancers, and then influence tumor-specific T cell immunity in a cancer micro-environment [13, 42, 70]. The host genetic status is likely to have an impact on the expected outcomes. In our meta-analysis, a total of 50 relevant publications were used to comprehensively assess relationships of PD-1/PD-L1 SNPs with cancer susceptibility. Our findings showed that PD-1.3 and PD-L1 rs17718883 were notably related to decreased cancer risk, while no significant associations were discovered in other PD-1 and PD-L1 SNPs. Differences in the genetic, ethnic background, cancer type and other baseline characteristics of the included subjects may be contributors to between-study heterogeneity. Therefore, subgroup analyses were further conducted to explore the source of heterogeneity.

Results indicated that PD-1.5 gene polymorphism was strongly linked with decreased risk of CC, NSCLC and OC. Inversely, the PD-1.5 variant significantly increased risk of GC, TC, Brain tumor, AML and UCC. There were remarkable associations of PD-1.9 SNP with BC, EC, OC and AML susceptibility. PD-1.3 A-allele mutant was dramatically related to lower BCC risk and increased CRC risk. As for PD-1.1, we detected significantly decreased associations in BC and OC risk. At the same time, PD-L1 rs4143815 variant remarkably decreased risk of GC, OC, and HCC, but elevated the BC susceptibility. We found that PD-L1 rs2890658 was slightly correlated with NSCLC, HCC and BC risk in some genetic models. The allelic, heterozygous and dominant models of PD-1.6 rs10204525 polymorphism have a positive association with AML risk. PD-L1 rs10815225 were prominently correlated with risk of GC, rs17718883 polymorphism with HCC and GC, and rs2297136 polymorphism with NSCLC and HCC, respectively. The PD-1.3 polymorphism was markedly correlated with lower cancer risk in certain subgroups of Asians, HB and higher quality score. PD-L1 rs10815225 C-allele significantly decreased the cancer risk among Asians. Similarly, PD-L1 rs17718883 polymorphism was notably associated with reduced cancer risk in Asians, HB, small sample size, lower and higher quality score subgroups.

The PD-1.5 polymorphism is located in exon 5 and serves as a synonymous variation that fails to alter the final amino acid structure of PD-1 protein. A silent mutation (Ala/Ala) probably roots in the substitution of C for T at +7785 position [85]. This significant association may attribute to this synonymous variant through linkage disequilibrium with other PD-1 gene polymorphisms, which influences PD-1 expression at mRNA and protein levels [86]. A study indicated that PD-1.5 CT genotype may render the risk of thyroid carcinoma by 2 times compared with CC/TT genotype among Italians [52]. The CT genotype might evidently enhanced risk of CC, GC, colon cancer, brain tumor and esophageal squamous cell carcinoma, suggesting PD- 1.5 variant as a risk factor in some cancers. A recent meta-analysis implied that harboring TT genotypes and T allele markedly decreased cancer risk [29]. The large variety in PD-1.5 genotype and allele frequency come from the molecular pathology, tumor location, and different ethnic groups. Interestingly, the PD-1.9 polymorphism is located at position +7625 in exon 5, which causes C to T substitution in extra cellular domain of PD-1 receptor during protein synthesis, affecting the sequence, resulting in alteration of structural and practical variations of PD-1 [29, 85]. For instance, variants in other undetected coinhibitory molecules, such as CD28 and lymphocyte activating 3 (LAG3) could affect the PD-1.9 function [76]. Additionally, PD-1.9 variant was found to be remarkably related to high expression of Her-2, showing that the genotype may regulate expression of oncogenes in occurrence and development of cancer [51].

PD-1.3 polymorphism is a guanine (G) to adenine (A) conversion at nucleotide +7146 position in intron 4. Given the existence of four tandem repeats containing some putative binding sequences of transcription factors, it was described as an enhancer-like structure [87, 88]. Previous studies have proved that the PD-1.3 polymorphism might influence binding of runt-related transcription factor 1 (RUNX1) and change transcriptional regulation and efficiency of PD-1 gene [87, 89]. Besides, the PD-1.3 A-allele could disrupt the binding site for RUNX1 transcription factors, which causes the impairment of PD-1 inhibitory effect and higher lymphocyte activity, thus enhancing tumor immunity capacity and reducing the liability of cancer cells [29, 90]. Consistently, the results showed an observably reduced cancer risk in heterozygous model of PD-1.3 polymorphism. Among these PD-1 gene polymorphisms, PD-1.1 is located at transcription start site or in the promoter region. It is well known that transcription initiation takes part in the regulation of gene expression. The variants in promoter region may interrupt the engagement between transcription factor binding sites (TFBS) and sequence motifs, and further affect the transcription start and activation of gene, increasing or decreasing the levels of mRNA and protein [91, 92]. It has been found that the frequency of PD-1.1 A-allele was higher in cases with p53 mutation, manifesting that expression of certain oncogenes could have superimposed effects with PD-1.1 polymorphism in cancer progression [36, 93]. Consistent with our results, Da et al. proved a negative correlation between the PD-1.1 SNP and cancer risk among Asians [94].

PD-1 rs7421861 polymorphism is located in the intron 1, where the multiple regulatory components and splicing control elements consist. The mutations in the region interrupt splicing sites, inhibit translation, and modify the secondary structure of mRNA [49]. Hence, the PD-1 rs7421861 variation may cause abnormal splicing and have impact on translational prevention [36, 44, 51]. Accumulative studies reported no remarkable correlation of the rs7421861 polymorphism with cancer risk [29]. Similarly, our results indicated no association between the rs7421861 and cancer predisposition. More importantly, the rs4143815 polymorphism is located near the 3’-untranslated region (3’-UTR). MicroRNAs (miRNAs) are a class of endogenous non-coding RNAs with 19-24 nucleotides that interact with complementary sites in the 3'-UTR of target mRNAs to regulate gene expression at post-transcriptional level [95]. Several miRNAs have been found to possess the potential ability to bind to the rs4143815 3'-UTR region, such as miR-570, miR-7-1, miR-495 and miR-298 [55]. In particular, studies verified that a G-to-C substitution in the rs4143815 locus could interrupt mRNA degradation mediated by miRNAs, and thus elevate the risk of cancer [42].

There are some limitations should be addressed in the present meta-analysis. First of all, there is little studies on PD-L1 rs10815225, rs17718883, and rs2297136 polymorphisms, leading to insufficient statistical power and decreased credibility of the results. Secondly, cancer is a multifactorial disease influenced by complex interactions between environmental exposure and genetic factors. Due to lack of sufficient data, other risk factors such as age, diet, smoking, heavy alcohol intake, obesity and family history of cancer were not evaluated in the meta-analysis. For instance, only one study provided the detailed distribution of PD-1.6, PD-1.1, and PD-L1 rs7421861 genotypes in esophageal cancer for gender, age, smoking, and alcohol variables [72]. Thirdly, almost all studies included in the meta-analysis mainly focused on the Asians in PD-1.3, PD-1.6, PD-L1 rs7421861, rs2890658, rs17718883 and rs2297136 polymorphisms. Therefore, large-scale studies in different ethnicity are considered to clarify the potential role of PD-1/PD-L1 in the progression of cancer.

## CONCLUSIONS

In conclusion, our results supported that PD-1.3 and PD-L1 rs17718883 were notably associated with lower cancer risk. The PD-1.5 mutant was remarkably correlated with CC, NSCLC, TC, Brain tumor, AML and UCC susceptibility. The PD-1.9 SNP markedly decreased risk of BC and AML, but increased risk of EC and OC. There was evident association of the PD-1.3 variant with CRC and BCC risk. Intriguingly, the PD-1.1 variant was slightly related to increased BC and OC risk. The rs4143815 was negatively associated with risk of GC, OC and HCC, but positively associated with risk of BC. PD-1.6 was prominently linked with AML risk, PD-L1 rs2890658 with NSCLC, HCC and BC risk, PD-L1 rs17718883 with HCC and GC risk, PD-L1 rs10815225 with GC risk, and PD-L1 rs2297136 with NSCLC and HCC risk. Importantly, PD-1 rs7421861, PD-L1 rs10815225, and rs10815225 polymorphisms dramatically reduced the risk of cancer among Asians, respectively. The rs7421861 notably decreased risk of cancer, while the rs10815225 elevated risk of cancers among Caucasians. To further confirm the findings, studies with large scale and well-matched controls from different ethnic groups are needed in the future.

## MATERIALS AND METHODS

This meta-analysis was performed according to the Preferred Reporting Items for Systematic Reviews and Meta-Analyses (PRISMA) statement [96]. All collected data were based on the previous published studies, and thus no ethical approval was required.

### Search strategy and study selection

A systematic literature retrieval was performed using the PubMed, EMBASE, and Cochrane Library data to obtain all relevant case-control studies published before 5 October, 2023. To determine the association between PD-1/PD-L1 variations and risk of cancer, we used the following terms: “programmed cell death 1 or programmed cell death ligand1 or PD-1 or PDCD1 or PD-L1” and “genotype or polymorphism or mutation or variant or variation or SNP” and “tumor or cancer or carcinoma or neoplasm” without any restriction on language and publication date. Besides, the reference lists of included studies were also screened by hand for the additional potential publications.

### Inclusion and exclusion criteria

All the studies included in the present analysis met the following criteria: (a) case-control studies; (b) evaluation of the associations between PD-1/PDL-1 gene polymorphisms and cancer risk; (c) containing available data for genotype frequencies; (d) sufficient information for evaluating ORs; sufficient data for calculating odds ratios (ORs) and 95% confidence intervals (CIs); (e) studies published only in English or Chinese. Accordingly, the exclusion criteria were as follows: (a) duplicate data; (b) case reports, comment, reviews, editorials, editorials, animal studies and conference papers; (c) short of complete genotype frequency data.

### Data extraction and quality assessment

Two participants (Yang and Liu) independently conducted literature screening, data extraction, and quality assessment. Any divergences could be fully resolved through discussion with a third investigator. The following information was collected from each publication: author’s name, publication year, country, ethnicity, cancer type, sample size of the participants, source of control*,* genotyping method, genotype distribution and *P*-value of HWE. Different ethnicities were stratified to Caucasian and Asian, and the study designs were categorized as population-based studies (PB) and hospital-based studies (HB).

The quality of each study was assessed in light of the Newcastle-Ottawa Scale (NOS), which included selection of study groups (4 stars), comparability of the groups (2 stars) and ascertain of exposure or outcome (3 stars) with a rating range of 0–9 stars [97]. According to the evaluation items, scores greater than 6 were considered high-quality literature. The higher the score, the better the quality.

### Statistical analysis

The strength of association of PD-1/PDL-1 gene polymorphism with cancer risk was appraised by crude ORs with corresponding 95% Cis. For each SNP, the pooled ORs were calculated in allelic, homozygote, heterozygote, dominant and recessive models, respectively. After that, Chi-square-based *Q* and *I*^2^ tests were utilized to determine the heterogeneity among included studies. When I^2^ < 50% and p ≥ 0.05, it indicated that there was no statistical heterogeneity, and the fixed-effect (FEM) model was selected for calculation. Otherwise, the random-effect model (REM) was applied. Subgroup analysis was performed to obtain more specific results on the basis of ethnicity, cancer types, sources of control, sample size of participants and quality score. In order to evaluate the robustness of the results, sensitivity analyses were conducted by sequentially excluding each study. Egger’s test and Begg’s funnel plots were used to judge the publication bias. If P < 0.05 indicates obvious publication bias. All data analyses were performed by the STATA software (Version 16.0; Stata Corporation, College Station, TX, USA).

### False-positive report probability (FPRP) analysis

The probability of meaningful associations of PD-1 and PD-L1 gene polymorphisms with cancer risk can be determined through conducting the FPRP analysis [98]. In order to investigate the evident relationships observed in this meta-analysis, we adopted prior probabilities of 0.25, 0.1, 0.01, 0.001, and 0.0001 and computed the FPRP values as described previously. The association that reached the FPRP threshold of < 0.2 was considered significant.

## Supplementary Material

Supplementary Figures

Supplementary Table 1

Supplementary Table 2

Supplementary Table 3
