# Supplementary material for: Associations of PD-1 and PD-L1 gene polymorphisms with cancer risk: a meta-analysis based on 50 studies
Source: Aging (Albany NY). 2024 Mar 27;16(7):6068–97. doi: 10.18632/aging.205689 (PMC11042937; doi:10.18632/aging.205689)
Supplement: Supplementary Figures [file aging-16-205689-s001.pdf]

SUPPLEMENTARY FIGURES

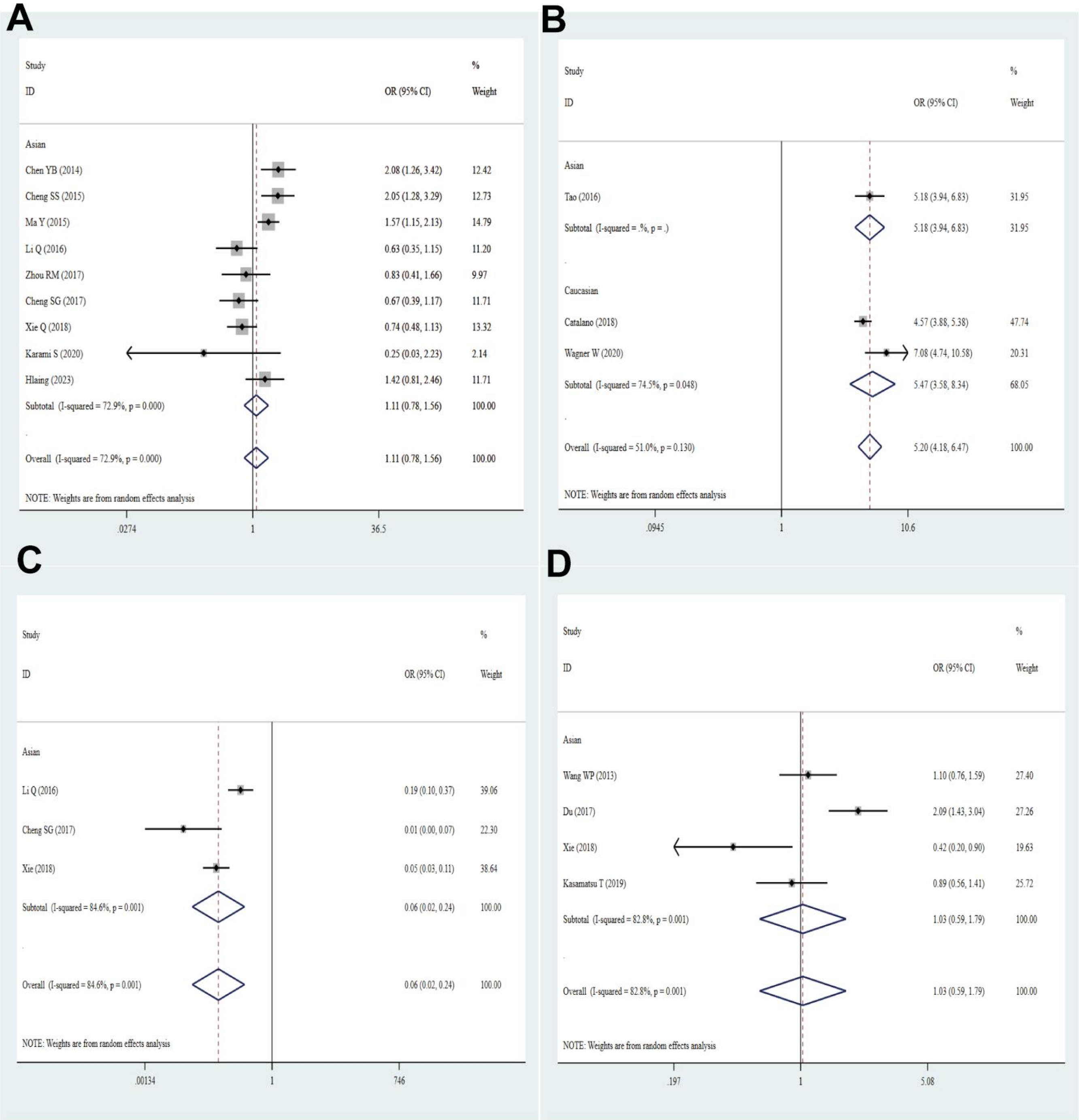

Supplementary Figure 1. Forest plot for the association between PD-L1 gene polymorphisms and cancer susceptibility in the homozygote model. For each publication, the estimation of OR and its 95% CI was plotted with a box and a horizontal line. The diamonds represented the pooled ORs and 95% CIs. (**A**) rs2890658 polymorphism; (**B**) rs10815225 polymorphism; (**C**) rs17718883 polymorphism; (**D**) rs2297136 polymorphism.

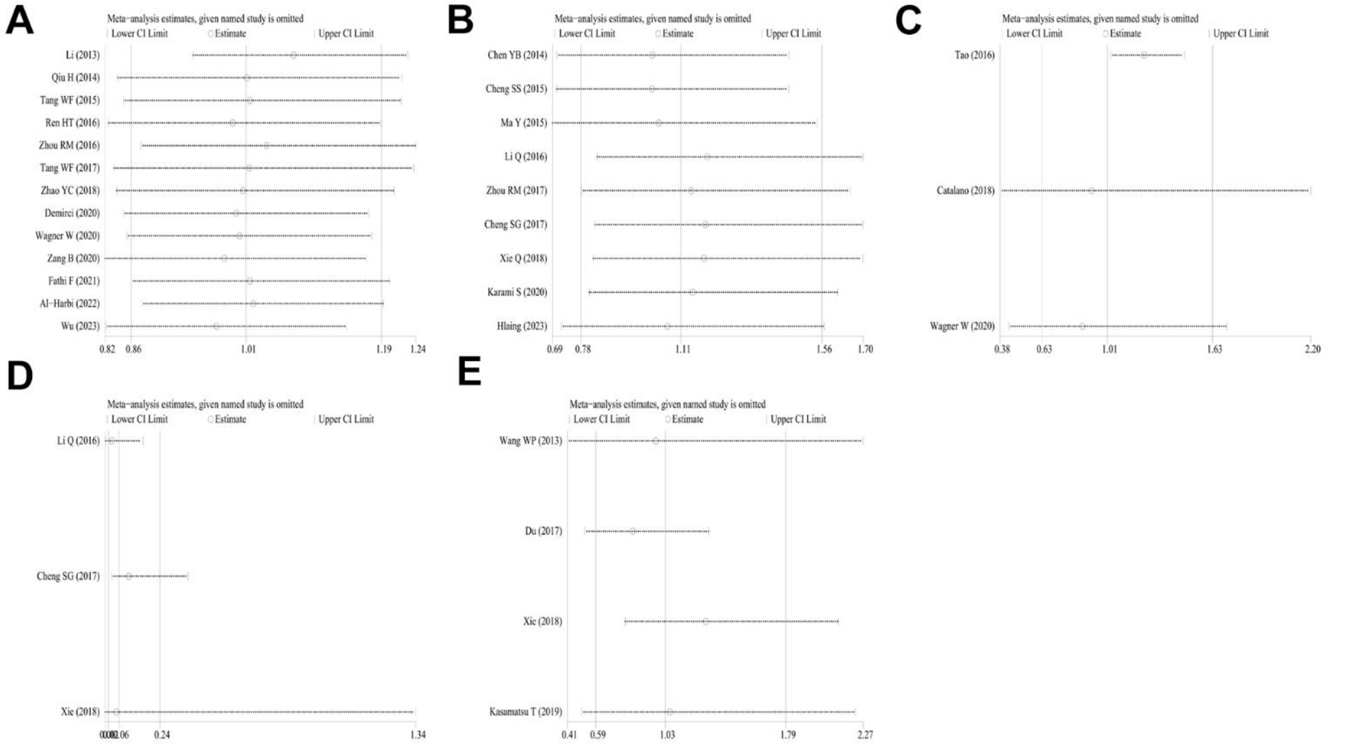

Supplementary Figure 2. Analyses of the influence of a single study on the total combined OR in the dominant model. (**A**) PD-1.6 polymorphism, (**B**) rs2890658 polymorphism; (**C**) rs10815225 polymorphism; (**D**) rs17718883 polymorphism; (**E**) rs2297136 polymorphism.

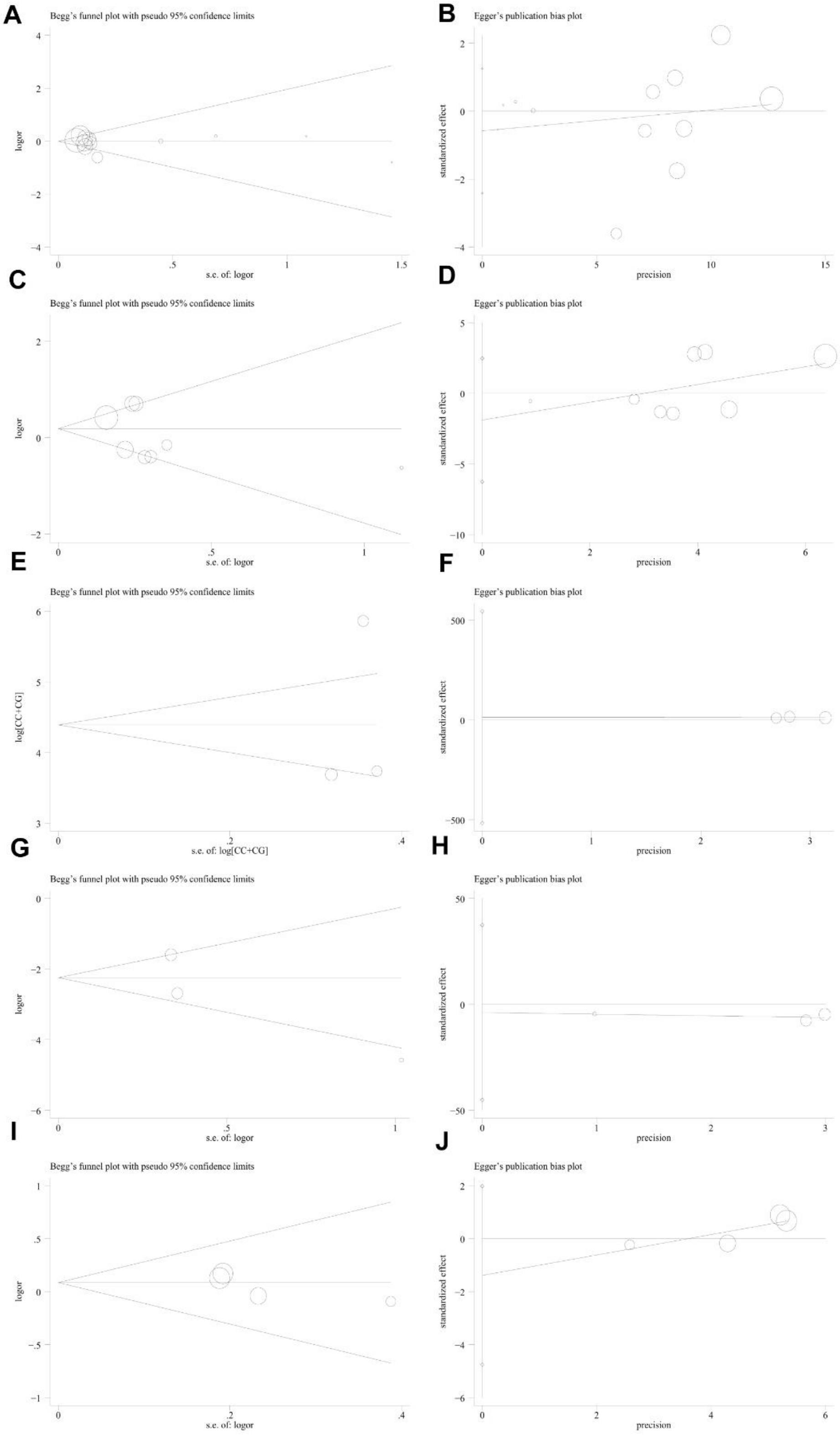

Supplementary Figure 3. Begg's funnel plot and Egger's linear regression test for the publication biases under the dominant model. (**A**) Begg's test for PD-1.6 polymorphism; (**B**) Egger's test for PD-1.6 polymorphism; (**C**) Begg's test for PD-L1 rs2890658 polymorphism; (**D**) Egger's test for PD-L1 rs2890658 polymorphism; (**E**) Begg's test for PD-L1 rs10815225 polymorphism; (**F**) Egger's test for PD-L1 rs10815225 polymorphism; (**G**) Begg's test for PD-L1 rs17718883 polymorphism; (**H**) Egger's test for PD-L1 rs17718883 polymorphism; (**I**) Begg's test for PD-L1 rs2297136 polymorphism; (**J**) Egger's test for PD-L1 rs2297136 polymorphism.
