# Supplementary material for: Associations of PD-1 and PD-L1 gene polymorphisms with cancer risk: a meta-analysis based on 50 studies
Source: Aging (Albany NY). 2024 Mar 27;16(7):6068–97. doi: 10.18632/aging.205689 (PMC11042937; doi:10.18632/aging.205689)
Supplement: Supplementary Table 1 [file aging-16-205689-s002.doc]

Supplementary Table 1. Distribution PD-1 and PD-L1 gene polymorphisms in multiple cancers.

| First author | Year | SNP | Case | | | |  | Control | | | |  | HWE |
| --- | --- | --- | --- | --- | --- | --- | --- | --- | --- | --- | --- | --- | --- |
|  |  |  | N | AA | Aa | aa |  | N | AA | Aa | aa |  | (P) |
| Emma L | 2010 | PD-1.5 | 1300 | 471 | 603 | 226 |  | 556 | 176 | 258 | 122 |  | 0.1368 |
| Haghshenas | 2011 | PD-1.3 | 436 | 365 | 63 | 8 |  | 290 | 231 | 55 | 4 |  | 0.7257 |
|  |  | PD-1.5 | 435 | 194 | 191 | 50 |  | 328 | 137 | 145 | 46 |  | 0.4455 |
| Hua Z | 2011 | PD-1.1 | 490 | 103 | 271 | 116 |  | 512 | 140 | 260 | 112 |  | 0.6727 |
|  |  | PD-1.5 | 486 | 295 | 169 | 22 |  | 478 | 244 | 210 | 24 |  | 0.0121^*^ |
|  |  | PD-1.9 | 487 | 111 | 249 | 127 |  | 506 | 95 | 268 | 143 |  | 0.1211 |
|  |  | rs7421861 | 490 | 333 | 146 | 11 |  | 512 | 370 | 130 | 12 |  | 0.8845 |
| Bayram S | 2012 | PD-1.3 | 236 | 191 | 45 | 0 |  | 236 | 180 | 56 | 0 |  | 0.0386^*^ |
| Mojtahedi Z | 2012 | PD-1.5 | 200 | 59 | 109 | 32 |  | 200 | 75 | 89 | 36 |  | 0.2900 |
| Li | 2013 | PD-1.6 | 271 | 180 | 83 | 8 |  | 318 | 160 | 130 | 28 |  | 0.8280 |
| Yousefi AR | 2013 | PD-1.3 | 80 | 18 | 27 | 35 |  | 100 | 43 | 35 | 22 |  | 0.0074^*^ |
| Savabkar S | 2013 | PD-1.5 | 122 | 50 | 66 | 6 |  | 166 | 89 | 70 | 7 |  | 0.0071^*^ |
| Wang WP | 2013 | rs2297136 | 205 | 142 | 49 | 14 |  | 393 | 280 | 96 | 17 |  | 0.0224^*^ |
|  |  | rs4143815 | 205 | 45 | 72 | 88 |  | 393 | 135 | 188 | 70 |  | 0.7459 |
| Chen YB | 2014 | PD-L1 8293 | 293 | 242 | 48 | 3 |  | 293 | 266 | 26 | 1 |  | 0.6708 |
| Qiu H | 2014 | PD-1.6 | 600 | 317 | 240 | 43 |  | 651 | 345 | 243 | 63 |  | 0.0387^*^ |
|  |  | PD-1.9 | 616 | 159 | 303 | 154 |  | 681 | 189 | 325 | 167 |  | 0.2453 |
|  |  | rs7421861 | 600 | 411 | 168 | 21 |  | 673 | 460 | 188 | 25 |  | 0.2945 |
| Yin L | 2014 | PD-1.5 | 324 | 198 | 106 | 20 |  | 330 | 181 | 105 | 44 |  | 0.0000^*^ |
| Cheng SS | 2015 | PD-L1 8293 | 288 | 233 | 51 | 4 |  | 300 | 269 | 30 | 1 |  | 0.3611 |
| Ge J | 2015 | rs7421861 | 596 | 395 | 187 | 14 |  | 620 | 440 | 163 | 17 |  | 0.6847 |
| Ma Y | 2015 | PD-1.1 -538 | 528 | 144 | 246 | 138 |  | 600 | 156 | 296 | 148 |  | 0.7472 |
|  |  | PD-1.3 7146 | 528 | 426 | 102 | 0 |  | 600 | 456 | 142 | 2 |  | 0.0086^*^ |
|  |  | PD-1.5 7785 | 528 | 244 | 216 | 68 |  | 600 | 256 | 246 | 98 |  | 0.0036^*^ |
|  |  | PD-1.9 7625 | 528 | 343 | 148 | 37 |  | 600 | 404 | 168 | 28 |  | 0.0565 |
|  |  | PD-L1 8293 | 528 | 416 | 106 | 6 |  | 600 | 512 | 84 | 4 |  | 0.7849 |
| Tang WF | 2015 | PD-1.6 | 313 | 169 | 123 | 21 |  | 581 | 309 | 219 | 53 |  | 0.1200 |
|  |  | PD-1.9 | 330 | 75 | 168 | 87 |  | 603 | 163 | 292 | 148 |  | 0.4478 |
|  |  | rs7421861 | 324 | 226 | 91 | 7 |  | 598 | 408 | 168 | 22 |  | 0.3680 |
| Li XF | 2016 | PD-1.5 | 256 | 45 | 167 | 44 |  | 250 | 62 | 101 | 87 |  | 0.0037^*^ |
| Ren HT | 2016 | PD-1.6 | 557 | 172 | 257 | 128 |  | 582 | 137 | 299 | 146 |  | 0.8797 |
|  |  | PD-1.9 | 559 | 257 | 248 | 54 |  | 582 | 291 | 240 | 51 |  | 0.5034 |
|  |  | rs7421861 | 560 | 341 | 196 | 23 |  | 580 | 347 | 205 | 28 |  | 0.7456 |
| Haghshenas | 2016 | PD-1.3 | 95 | 82 | 13 | 0 |  | 160 | 127 | 30 | 3 |  | 0.4401 |
| Haghshenas | 2016 | PD-1.5 | 105 | 40 | 51 | 14 |  | 160 | 99 | 51 | 10 |  | 0.3309 |
| Zhou RM | 2016 | PD-1.1 | 584 | 147 | 303 | 134 |  | 585 | 145 | 298 | 142 |  | 0.6488 |
|  |  | PD-1.5 | 584 | 291 | 241 | 52 |  | 585 | 310 | 229 | 46 |  | 0.6832 |
|  |  | PD-1.6 | 584 | 325 | 226 | 33 |  | 585 | 296 | 238 | 51 |  | 0.7486 |
|  |  | PD-1.9 | 584 | 149 | 305 | 130 |  | 585 | 150 | 297 | 138 |  | 0.7021 |
| Li Q | 2016 | rs4143815 | 101 | 41 | 47 | 13 |  | 141 | 49 | 76 | 16 |  | 0.0953 |
|  |  | PD-L1 8293 | 101 | 79 | 20 | 2 |  | 141 | 98 | 39 | 4 |  | 0.9597 |
|  |  | rs17718883 | 101 | 87 | 13 | 1 |  | 141 | 77 | 48 | 16 |  | 0.0538 |
| Tao | 2016 | rs10815225 | 350 | 310 | 38 | 2 |  | 500 | 411 | 89 | 0 |  | 0.0289^*^ |
|  |  | rs4143815 | 346 | 70 | 153 | 123 |  | 500 | 160 | 223 | 117 |  | 0.0234^*^ |
| Du | 2017 | rs4143815 | 320 | 123 | 145 | 52 |  | 199 | 79 | 80 | 40 |  | 0.0208^*^ |
|  |  | rs2297136A/G | 320 | 83 | 226 | 11 |  | 199 | 84 | 100 | 15 |  | 0.0446^*^ |
|  |  | rs4742098A/G | 320 | 67 | 189 | 64 |  | 199 | 51 | 90 | 58 |  | 0.1832 |
| Zhou RM | 2017 | rs4143815 | 575 | 211 | 277 | 87 |  | 577 | 203 | 289 | 85 |  | 0.2749 |
|  |  | PD-L1 8293 | 575 | 18 | 161 | 396 |  | 577 | 15 | 144 | 418 |  | 0.5406 |
| Jahromi | 2017 | PD-1.3 | 152 | 130 | 19 | 3 |  | 150 | 116 | 30 | 4 |  | 0.2396 |
|  |  | PD-1.5 | 152 | 65 | 71 | 16 |  | 150 | 94 | 47 | 9 |  | 0.3461 |
| Li Y | 2017 | PD-1.1 | 620 | 150 | 301 | 169 |  | 620 | 168 | 323 | 129 |  | 0.2513 |
|  |  | PD-1.5 | 620 | 351 | 233 | 36 |  | 620 | 319 | 250 | 51 |  | 0.8374 |
| Tan D | 2017 | PD-1.9 | 164 | 87 | 60 | 17 |  | 170 | 111 | 48 | 11 |  | 0.0751 |
|  |  | rs4143815 | 164 | 31 | 82 | 51 |  | 170 | 54 | 78 | 38 |  | 0.3336 |
| Tang WF | 2017 | PD-1.1 | 1041 | 238 | 521 | 282 |  | 1674 | 430 | 800 | 444 |  | 0.0709 |
|  |  | PD-1.6 | 1039 | 544 | 397 | 98 |  | 1674 | 870 | 672 | 132 |  | 0.8879 |
|  |  | PD-1.9 | 1041 | 220 | 549 | 272 |  | 1674 | 416 | 816 | 442 |  | 0.3092 |
|  |  | rs7421861 | 1041 | 642 | 358 | 41 |  | 1674 | 1166 | 454 | 54 |  | 0.2320 |
| Cheng SG | 2017 | PD-L1 8293 | 123 | 95 | 27 | 1 |  | 141 | 98 | 39 | 4 |  | 0.9597 |
|  |  | rs4143815 | 123 | 50 | 50 | 23 |  | 141 | 49 | 76 | 16 |  | 0.0953 |
|  |  | rs17718883 | 123 | 122 | 1 | 0 |  | 141 | 77 | 48 | 16 |  | 0.0538 |
| Wei L | 2017 | PD-1.1 | 116 | 22 | 57 | 37 |  | 110 | 36 | 53 | 21 |  | 0.8494 |
|  |  | PD-1.5 | 116 | 67 | 42 | 7 |  | 110 | 60 | 44 | 6 |  | 0.5711 |
| Catalano | 2018 | rs4143815 | 1360 | 148 | 580 | 632 |  | 1103 | 122 | 467 | 514 |  | 0.3063 |
|  |  | rs10815225 | 1355 | 1003 | 340 | 12 |  | 1099 | 850 | 237 | 12 |  | 0.3136 |
|  |  | rs822338T/C | 1350 | 703 | 528 | 119 |  | 1100 | 561 | 435 | 104 |  | 0.1436 |
|  |  | rs866066C/T | 1344 | 414 | 632 | 298 |  | 1094 | 302 | 537 | 255 |  | 0.5860 |
|  |  | rs2890657GC | 1351 | 825 | 452 | 74 |  | 1100 | 667 | 368 | 65 |  | 0.1371 |
| Pirdelkhosh | 2018 | PD-1.3 | 206 | 171 | 31 | 4 |  | 173 | 144 | 26 | 3 |  | 0.1685 |
|  |  | PD-1.5 | 206 | 78 | 100 | 28 |  | 173 | 60 | 89 | 24 |  | 0.3209 |
| Zhao YC | 2018 | PD-1.1 | 419 | 96 | 207 | 116 |  | 497 | 121 | 253 | 123 |  | 0.6862 |
| Zhao YC | 2018 | PD-1.5 | 404 | 237 | 139 | 28 |  | 487 | 257 | 195 | 35 |  | 0.8102 |
|  |  | PD-1.6 | 410 | 204 | 169 | 37 |  | 488 | 249 | 204 | 35 |  | 0.4378 |
|  |  | PD-1.9 | 412 | 97 | 201 | 114 |  | 499 | 119 | 258 | 122 |  | 0.4461 |
| Shamsdin | 2018 | PD.1.1 | 76 | 60 | 15 | 1 |  | 73 | 18 | 28 | 27 |  | 0.0355^*^ |
| Gabriela V | 2018 | PD-1.1 | 250 | 226 | 18 | 6 |  | 250 | 225 | 25 | 0 |  | 0.4053 |
|  |  | PD-1.5 | 250 | 87 | 126 | 37 |  | 250 | 85 | 130 | 35 |  | 0.1876 |
|  |  | PD-1.9 | 250 | 227 | 21 | 2 |  | 250 | 225 | 25 | 0 |  | 0.4053 |
|  |  | rs41386349C/T | 250 | 229 | 21 | 0 |  | 250 | 213 | 34 | 3 |  | 0.2290 |
| Fathi F | 2018 | PD-1.3 | 150 | 119 | 27 | 4 |  | 150 | 113 | 32 | 5 |  | 0.1624 |
|  |  | PD-1.5 | 150 | 65 | 69 | 16 |  | 150 | 66 | 71 | 13 |  | 0.3169 |
|  |  | PD-1.9 | 150 | 146 | 4 | 0 |  | 150 | 146 | 4 | 0 |  | 0.8685 |
| Xie | 2018 | rs4143815 | 225 | 50 | 101 | 74 |  | 200 | 65 | 104 | 31 |  | 0.3157 |
|  |  | rs2297136 | 225 | 126 | 74 | 25 |  | 200 | 128 | 62 | 10 |  | 0.4889 |
|  |  | PD-L1 8293 | 225 | 170 | 49 | 6 |  | 200 | 139 | 55 | 6 |  | 0.8437 |
|  |  | rs17718883 | 225 | 215 | 8 | 2 |  | 200 | 108 | 69 | 23 |  | 0.0256^*^ |
| Kasamatsu T | 2019 | PD-1.1 | 124 | 37 | 54 | 33 |  | 211 | 51 | 110 | 50 |  | 0.5353 |
|  |  | PD-1.9 | 124 | 43 | 55 | 26 |  | 211 | 55 | 116 | 40 |  | 0.1268 |
|  |  | rs4143815 | 124 | 40 | 59 | 25 |  | 211 | 53 | 116 | 42 |  | 0.1364 |
|  |  | rs41386349C/T | 124 | 74 | 40 | 10 |  | 211 | 122 | 79 | 10 |  | 0.5364 |
|  |  | rs2297136 | 124 | 78 | 39 | 7 |  | 211 | 127 | 74 | 10 |  | 0.8520 |
| Fathi F | 2019 | PD-1.3 | 210 | 183 | 24 | 3 |  | 320 | 255 | 58 | 7 |  | 0.0986 |
|  |  | PD-1.5 | 210 | 93 | 87 | 30 |  | 320 | 150 | 134 | 36 |  | 0.4659 |
| Ramzi | 2020 | PD-1.3 | 59 | 38 | 18 | 3 |  | 38 | 21 | 13 | 4 |  | 0.3728 |
|  |  | PD-1.9 | 59 | 38 | 18 | 3 |  | 38 | 17 | 19 | 2 |  | 0.4659 |
| Karami S | 2020 | PD-1.3 | 260 | 234 | 26 | 0 |  | 260 | 245 | 15 | 0 |  | 0.6320 |
|  |  | PD-1.5 | 260 | 113 | 139 | 8 |  | 260 | 130 | 125 | 5 |  | 0.0001^*^ |
|  |  | PD-1.9 | 260 | 211 | 47 | 2 |  | 260 | 200 | 56 | 4 |  | 0.9718 |
|  |  | rs4143815 | 260 | 79 | 161 | 20 |  | 260 | 84 | 135 | 41 |  | 0.2752 |
|  |  | PD-L1 8293 | 260 | 4 | 155 | 101 |  | 260 | 1 | 100 | 159 |  | 0.0004^*^ |
| Demirci | 2020 | PD-1.1 | 137 | 124 | 13 | 0 |  | 136 | 123 | 13 | 0 |  | 0.5583 |
|  |  | PD-1.5 | 137 | 66 | 55 | 16 |  | 136 | 56 | 67 | 13 |  | 0.2693 |
|  |  | PD-1.6 | 137 | 3 | 38 | 96 |  | 136 | 8 | 35 | 93 |  | 0.0700 |
| Wagner W | 2020 | PD-1.1 | 208 | 201 | 7 | 0 |  | 256 | 249 | 7 | 0 |  | 0.8245 |
|  |  | PD-1.3 | 260 | 234 | 26 | 0 |  | 260 | 245 | 15 | 0 |  | 0.4452 |
|  |  | PD-1.5 | 208 | 70 | 98 | 40 |  | 256 | 82 | 132 | 42 |  | 0.3613 |
|  |  | PD-1.6 | 208 | 1 | 35 | 172 |  | 256 | 6 | 42 | 208 |  | 0.0368^*^ |
|  |  | rs7421861 | 208 | 96 | 81 | 31 |  | 256 | 99 | 129 | 28 |  | 0.1419 |
| Wagner W | 2020 | rs4143815 | 205 | 97 | 82 | 26 |  | 252 | 122 | 103 | 27 |  | 0.4545 |
|  |  | rs10815225 | 205 | 163 | 42 | 0 |  | 252 | 214 | 34 | 4 |  | 0.0635 |
|  |  | rs822335C/T | 205 | 77 | 105 | 23 |  | 252 | 102 | 112 | 38 |  | 0.4290 |
|  |  | rs4742098A/G | 205 | 122 | 71 | 12 |  | 252 | 137 | 99 | 16 |  | 0.7372 |
| Zang B | 2020 | PD-1.1 | 813 | 673 | 132 | 8 |  | 961 | 761 | 188 | 12 |  | 0.9194 |
|  |  | PD-1.6 | 812 | 420 | 329 | 63 |  | 960 | 551 | 359 | 50 |  | 0.3881 |
|  |  | rs7421861 | 813 | 100 | 370 | 343 |  | 960 | 92 | 411 | 457 |  | 0.9766 |
| Fathi F | 2021 | PD-1.1 | 210 | 151 | 50 | 9 |  | 220 | 165 | 47 | 8 |  | 0.0551 |
|  |  | PD-1.6 | 210 | 11 | 56 | 143 |  | 220 | 10 | 62 | 148 |  | 0.2942 |
| Cevik M | 2021 | PD-1.1 | 77 | 69 | 8 | 0 |  | 44 | 21 | 23 | 0 |  | 0.0308^*^ |
|  |  | PD-1.9 | 82 | 76 | 6 | 0 |  | 77 | 73 | 4 | 0 |  | 0.8150 |
|  |  | rs2282055T/G | 79 | 44 | 30 | 5 |  | 76 | 32 | 34 | 10 |  | 0.0171^*^ |
|  |  | rs822336G/C | 80 | 21 | 42 | 17 |  | 76 | 25 | 37 | 14 |  | 0.9619 |
| Al-Harbi | 2022 | PD-1.5 | 100 | 18 | 30 | 52 |  | 100 | 8 | 40 | 52 |  | 0.9367 |
|  |  | PD-1.6 | 100 | 9 | 20 | 71 |  | 100 | 0 | 19 | 81 |  | 0.2938 |
|  |  | PD-1.9 | 100 | 38 | 57 | 5 |  | 100 | 40 | 56 | 4 |  | 0.0041* |
| Wu | 2023 | PD-1.5 | 285 | 134 | 124 | 27 |  | 324 | 180 | 114 | 30 |  | 0.0604 |
|  |  | PD-1.9 | 285 | 88 | 176 | 21 |  | 324 | 85 | 164 | 75 |  | 0.8106 |
|  |  | PD-1.6 | 285 | 115 | 138 | 32 |  | 324 | 164 | 132 | 28 |  | 0.8442 |
| Katarzyna | 2023 | PD-1.5 | 30 | 10 | 15 | 5 |  | 30 | 8 | 13 | 9 |  | 0.4684 |
|  |  | PD-1.3 | 30 | 23 | 5 | 2 |  | 30 | 29 | 1 | 0 |  | 0.9260 |
| Hlaing | 2023 | PD-1.5 | 256 | 134 | 104 | 8 |  | 211 | 130 | 68 | 13 |  | 0.3143 |
|  |  | PD-L1 8293 | 256 | 218 | 36 | 2 |  | 211 | 187 | 22 | 1 |  | 0.6873 |

AA: homozygote; Aa: common heterozygote; aa: rare homozygote. ^*^P<0.05.
