# Supplementary material for: Associations of PD-1 and PD-L1 gene polymorphisms with cancer risk: a meta-analysis based on 50 studies
Source: Aging (Albany NY). 2024 Mar 27;16(7):6068–97. doi: 10.18632/aging.205689 (PMC11042937; doi:10.18632/aging.205689)
Supplement: Supplementary Table 2 [file aging-16-205689-s003.pdf]

SUPPLEMENTARY TABLE

Supplementary Table 2. Quality assessment of included studies according to the Newcastle-Ottawa Scale.

		Selection				Comparability	Exposure			
**Author**	**Year**	**Adequate definition of case**	**Representative of the cases**	**Selection of controls**	**Definition of controls**	**Comparability of cases and controls on the basis of the design or analysis**	**Ascertainment of exposure**	**Same method of ascertainment for cases and controls**	**Non-response rates**	**NOS**
Emma L	2010	☆	☆	☆	☆	☆	☆	☆		7
Haghshenas	2011	☆	☆	☆	☆	☆☆	☆	☆		8
Hua Z	2011	☆	☆	☆	☆	☆☆	☆	☆		8
Bayram S	2012	☆	☆		☆	☆☆	☆	☆		7
Mojtahedi Z	2012	☆	☆	☆	☆	☆	☆	☆		7
Li	2013	☆	☆	☆	☆	☆☆	☆	☆		8
Yousefi AR	013	☆	☆		☆	☆	☆	☆		6
Savabkar S	2013	☆	☆		☆	☆☆	☆	☆		7
Wang WP	2013	☆	☆		☆	☆	☆	☆		6
Chen YB	2014	☆	☆		☆	☆☆	☆	☆		7
Qiu H	2014	☆	☆		☆	☆☆	☆	☆		7
Yin L	2014	☆	☆	☆	☆	☆☆	☆	☆		8
Cheng SS	2015	☆	☆		☆	☆	☆	☆		6
Ge J	2015	☆	☆		☆	☆☆	☆	☆		7
Ma Y	2015	☆	☆	☆	☆	☆☆	☆	☆	☆	9
Tang WF	2015	☆	☆		☆	☆☆	☆	☆		7
Li XF	2016	☆	☆	☆	☆	☆☆	☆	☆		8
Ren HT	2016	☆	☆	☆	☆	☆☆	☆	☆		8
Haghshenas	2016	☆	☆	☆	☆	☆☆	☆	☆		8
Zhou RM	2016	☆	☆	☆	☆	☆☆	☆	☆	☆	9
Li Q	2016	☆	☆		☆	☆☆	☆	☆		7
Tao	2016	☆	☆		☆	☆	☆	☆		6
Du	2017	☆	☆		☆	☆☆	☆	☆		7
Zhou RM	2017	☆	☆	☆	☆	☆☆	☆	☆	☆	9
Jahromi	2017	☆	☆	☆	☆	☆☆	☆	☆		8
Li Y	2017	☆	☆		☆	☆☆	☆	☆		7
Tan D	2017	☆	☆	☆	☆	☆☆	☆	☆	☆	9
Tang WF	2017	☆	☆		☆	☆☆	☆	☆		7
Cheng SG	2017	☆	☆		☆	☆☆	☆	☆	☆	8
Wei L	2017	☆	☆		☆	☆☆	☆	☆		7
Catalano	2018	☆	☆		☆	☆☆	☆	☆		7
Pirdelkhosh	2018	☆	☆	☆	☆	☆☆	☆	☆		8
Zhao YC	2018	☆	☆		☆	☆	☆	☆		6
Shamsdin	2018	☆	☆		☆	☆	☆	☆		6
Gabriela V	2018	☆	☆	☆	☆	☆☆	☆	☆		8
Fathi F	2018	☆	☆		☆	☆	☆	☆		6
Xie	2018	☆	☆		☆	☆☆	☆	☆		7
Kasamatsu T	2019	☆	☆	☆	☆	☆	☆	☆		7
Fathi F	2019	☆	☆		☆	☆☆	☆	☆		7
Ramzi	2020	☆	☆		☆	☆	☆	☆		6
Karami S	2020	☆	☆		☆	☆	☆	☆		6
Demirci	2020	☆	☆		☆	☆☆	☆	☆		7
Wagner W	2020	☆	☆	☆	☆	☆☆	☆	☆	☆	9
Zang B	2020	☆	☆	☆	☆	☆☆	☆	☆	☆	9
Fathi F	2021	☆	☆		☆	☆	☆	☆		6
Cevik M	2021	☆	☆		☆	☆	☆	☆		6
Al-Harbi	2022	☆	☆		☆	☆☆	☆	☆		7
Wu	2023	☆	☆		☆	☆☆	☆	☆		7
Katarzyna	2023	☆	☆		☆	☆	☆	☆		6
Hlaing	2023	☆	☆		☆	☆☆	☆	☆		7
