# Supplementary material for: Associations of PD-1 and PD-L1 gene polymorphisms with cancer risk: a meta-analysis based on 50 studies
Source: Aging (Albany NY). 2024 Mar 27;16(7):6068–97. doi: 10.18632/aging.205689 (PMC11042937; doi:10.18632/aging.205689)
Supplement: Supplementary Table 3 [file aging-16-205689-s004.doc]

Supplementary Table 3. Stratified analyses of PD-1/PD-L1 gene polymorphisms with cancer risk.

| Locus | No. | Allele | | Homozygote | | Heterozygote | | Dominant | | Recessive | |
| --- | --- | --- | --- | --- | --- | --- | --- | --- | --- | --- | --- |
|  |  | OR (95%CI) *P* | I^2^ (%) | OR (95%CI) *P* | I^2^ (%) | OR (95%CI) *P* | I^2^ (%) | OR (95%CI) *P* | I^2^ (%) | OR (95%CI) *P* | I^2^ (%) |
| PD-1.5 rs2227981C/T | | | | | | | | | | | |
| Caucasian | 4 | 0.90 (0.79, 1.02) 0.098 | 13.7 | 0.83 (0.62, 1.11) 0.207 | 24.3 | 0.89 (0.75, 1.06) 0.184 | 0.0 | 0.86 (0.73, 1.01) 0.073 | 0.0 | 0.90 (0.66, 1.21) 0.469 | 38.1 |
| Asian | 21 | 1.01 (0.91, 1.12) 0.826 | 69.7 | 0.94 (0.78, 1.14) 0.537 | 47.8 | 1.10 (0.94, 1.29) 0.231 | 73.5 | 1.07 (0.92, 1.24) 0.366 | 73.0 | 0.90 (0.75, 1.09) 0.276 | 50.4 |
| Source of control |  |  |  |  |  |  |  |  |  |  |  |
| PB | 13 | 0.99 (0.86, 1.13) 0.832 | 76.4 | 0.92 (0.73, 1.16) 0.474 | 60.6 | 1.11 (0.92, 1.35) 0.283 | 76.2 | 1.06 (0.88, 1.28) 0.526 | 76.4 | 0.86 (0.69, 1.08) 0.195 | 64.9 |
| HB | 12 | 1.01 (0.90, 1.13) 0.862 | 42.0 | 0.93 (0.74, 1.16) 0.519 | 10.8 | 1.02 (0.84, 1.24) 0.835 | 60.2 | 1.02 (0.85, 1.22) 0.834 | 58.3 | 0.97 (0.80, 1.17) 0.724 | 0.0 |
| NOS scores |  |  |  |  |  |  |  |  |  |  |  |
| N1 | 14 | 0.99 (0.90, 1.10) 0.909 | 48.1 | 0.89 (0.73, 1.07) 0.216 | 18.2 | 1.04 (0.87, 1.23) 0.692 | 60.5 | 1.02 (0.87, 1.20) 0.814 | 60.2 | 0.88 (0.76, 1.01) 0.076 | 0.0 |
| N2 | 11 | 1.00 (0.85, 1.17) 0.975 | 78.4 | 0.95 (0.72, 1.25) 0.703 | 63.2 | 1.12 (0.89, 1.40) 0.337 | 77.8 | 1.07 (0.87, 1.32) 0.529 | 77.9 | 0.89 (0.67, 1.18) 0.424 | 70.2 |
| Sample size |  |  |  |  |  |  |  |  |  |  |  |
| S1 | 19 | 1.01 (0.89, 1.13) 0.909 | 67.4 | 0.98 (0.79, 1.22) 0.863 | 44.8 | 1.07 (0.89, 1.29) 0.482 | 72.2 | 1.05 (0.88, 1.25) 0.579 | 70.9 | 0.96 (0.77, 1.18) 0.673 | 53.3 |
| S2 | 6 | 0.96 (0.84, 1.11) 0.581 | 67.4 | 0.81 (0.64, 1.02) 0.067 | 38.5 | 1.05 (0.88, 1.26) 0.589 | 62.4 | 1.01 (0.84, 1.21) 0.947 | 68.4 | 0.80 (0.68, 0.95) 0.010 | 8.3 |
| Cancer type |  |  |  |  |  |  |  |  |  |  |  |
| CC | 2 | 0.83 (0.73, 0.93) 0.002^*^ | 0.0 | 0.69 (0.54, 0.89) 0.004^*^ | 0.0 | 1.38 (0.54, 3.53) 0.501 | 92.6 | 1.09 (0.58, 2.04) 0.785 | 85.3 | 0.55 (0.29, 1.05) 0.069 | 85.8 |
| BC | 4 | 0.90 (0.71, 1.13) 0.354 | 61.4 | 0.80 (0.57, 1.12) 0.195 | 0.0 | 0.91 (0.65, 1.27) 0.584 | 66.0 | 0.89 (0.64, 1.23) 0.490 | 67.1 | 0.84 (0.61, 1.16) 0.294 | 0.0 |
| CRC | 3 | 0.93 (0.75, 1.14) 0.464 | 35.0 | 0.84 (0.54, 1.31) 0.448 | 29.2 | 0.82 (0.42, 1.61) 0.568 | 82.1 | 0.85 (0.48, 1.49) 0.562 | 77.9 | 0.94 (0.69, 1.28) 0.689 | 0.0 |
| GC | 1 | 1.39 (0.96, 2.00) 0.079 | _ | 1.53 (0.49, 4.79) 0.469 | _ | 1.68 (1.04, 2.72) 0.036^*^ | _ | 1.66 (1.04, 2.67) 0.035^*^ | _ | 1.18 (0.39, 3.59) 0.777 | _ |
| NSCLC | 3 | 0.83 (0.72, 0.95) 0.009^*^ | 16.3 | 0.65 (0.44, 0.97) 0.036^*^ | 46.6 | 0.91 (0.76, 1.10) 0.324 | 0.0 | 0.84 (0.71, 0.99) 0.043^*^ | 0.0 | 0.69 (0.45, 1.04) 0.079 | 55.6 |
| TC | 1 | 2.12 (1.44, 3.11) 0.000^*^ | _ | 3.47 (1.42, 8.44) 0.006^*^ | _ | 2.48 (1.45, 4.22) 0.001^*^ | _ | 2.64 (1.59, 4.38) 0.000^*^ | _ | 2.31 (0.98, 5.41) 0.054 | _ |
| EC | 1 | 1.11 (0.93, 1.33) 0.260 | _ | 1.20 (0.79, 1.85) 0.394 | _ | 1.12 (0.88, 1.43) 0.353 | _ | 1.14 (0.90, 1.43) 0.279 | _ | 1.84 (0.79, 4.31) 0.521 | _ |
| Brain tumor | 1 | 1.85 (1.29, 2.66) 0.001^*^ | _ | 2.57 (1.07, 6.17) 0.035^*^ | _ | 2.19 (1.34, 3.55) 0.001^*^ | _ | 2.25 (1.42, 3.56) 0.001^*^ | _ | 1.84 (0.79, 4.31) 0.159 | _ |
| OC | 2 | 0.84 (0.71, 0.99) 0.036^*^ | 0.0 | 0.69 (0.45, 1.04) 0.079 | 0.0 | 0.85 (0.68, 1.05) 0.134 | 0.0 | 0.82 (0.67, 1.01) 0.062 | 0.0 | 0.73 (0.49, 1.11) 0.140 | 0.0 |
| Melanoma | 1 | 1.00 (0.78, 1.29) 1.000 | _ | 1.03 (0.60, 1.79) 0.908 | _ | 0.95 (0.64, 1.39) 0.782 | _ | 0.97 (0.67, 1.40) 0.851 | _ | 1.07 (0.65, 1.76) 0.799 | _ |
| HNSCC | 1 | 1.06 (0.76, 1.49) 0.728 | _ | 1.25 (0.56, 2.80) 0.589 | _ | 0.99 (0.61, 1.59) 0.956 | _ | 1.03 (0.65, 1.62) 0.907 | _ | 1.26 (0.58, 2.72) 0.558 | _ |
| BCC | 1 | 1.13 (0.88, 1.47) 0.342 | _ | 1.34 (0.78, 2.33) 0.291 | _ | 1.05 (0.42, 1.15) 0.809 | _ | 1.11 (0.78,1.58) 0.559 | _ | 1.32 (0.78, 2.21) 0.302 | _ |
| HCC | 1 | 0.90 (0.63, 1.28) 0.544 | _ | 1.04 (0.46, 2.36) 0.917 | _ | 0.70 (0.42, 1.15) 0.160 | _ | 0.75 (0.47, 1.22) 0.245 | _ | 1.25 (0.58, 2.71) 0.570 | _ |
| RCC | 1 | 1.03 (0.79, 1.33) 0.854 | _ | 1.12 (0.65, 1.91) 0.690 | _ | 0.87 (0.58, 1.31) 0.507 | _ | 0.93 (0.63, 1.37) 0.711 | _ | 1.21 (0.75, 1.96) 0.428 | _ |
| AML | 1 | 1.24 (0.97, 1.59) 0.093 | _ | 1.21 (0.69, 2.13) 0.511 | _ | 1.46 (1.04, 2.05) 0.028^*^ | _ | 1.41 (1.02, 1.94) 0.036^*^ | _ | 1.03 (0.59, 1.77) 0.928 | _ |
| UCC | 1 | 1.13 (0.83, 1.53) 0.452 | _ | 0.60 (0.24, 1.49) 0.268 | _ | 1.48 (1.01, 2.19) 0.047^*^ | _ | 1.34 (0.92, 1.95) 0.124 | _ | 0.51 (0.21, 1.26) 0.145 | _ |
| PD-1.9 rs2227982C/T | | | | | | | | | | | |
| Ethnicity |  |  |  |  |  |  |  |  |  |  |  |
| Caucasian | 1 | 1.00 (0.57, 1.77) 0.827 | _ | 4.96 (0.23, 103.81) 0.302 | _ | 0.83 (0.45, 1.53) 0.555 | _ | 0.91 (0.50, 1.65) 0.761 | _ | 5.04 (0.24, 105.52) 0.297 | _ |
| Asian | 16 | 0.98 (0.89, 1.07) 0.602 | 59.2 | 0.95 (0.77, 1.18) 0.641 | 66.2 | 0.98 (0.86, 1.12) 0.809 | 47.2 | 0.98 (0.85, 1.12) 0.722 | 55.1 | 0.96 (0.81, 1.14) 0.659 | 61.9 |
| Source of control |  |  |  |  |  |  |  |  |  |  |  |
| PB | 7 | 0.98 (0.84, 1.15) 0.831 | 63.2 | 0.99 (0.73, 1.33) 0.934 | 52.2 | 0.90 (0.72, 1.13) 0.372 | 59.8 | 0.93 (0.73, 1.17) 0.512 | 67.1 | 0.98 (0.84, 1.13) 0.734 | 13.6 |
| HB | 10 | 0.98 (0.87, 1.10) 0.696 | 53.7 | 0.92 (0.67, 1.26) 0.584 | 72.5 | 1.07 (0.93, 1.22) 0.347 | 18.3 | 1.03 (0.90, 1.19) 0.646 | 27.4 | 1.04 (0.93, 1.17) 0.477 | 75.3 |
| NOS scores |  |  |  |  |  |  |  |  |  |  |  |
| N1 | 11 | 0.96 (0.85, 1.08) 0.521 | 54.5 | 0.91 (0.67, 1.24) 0.550 | 68.9 | 1.02 (0.87, 1.19) 0.815 | 35.7 | 1.00 (0.85, 1.16) 0.945 | 39.5 | 1.04 (0.93, 1.17) 0.479 | 71.7 |
| N2 | 6 | 1.01 (0.86, 1.17) 0.941 | 64.8 | 1.00 (0.73, 1.38) 0.979 | 59.9 | 0.94 (0.76, 1.17) 0.575 | 57.9 | 0.97 (0.77, 1.22) 0.769 | 67.2 | 0.98 (0.84, 1.13) 0.736 | 28.0 |
| Sample size |  |  |  |  |  |  |  |  |  |  |  |
| S1 | 10 | 1.04 (0.91, 1.19) 0.543 | 25.1 | 1.18 (0.94, 1.48) 0.153 | 0.0 | 0.96 (0.77, 1.19) 0.691 | 39.1 | 0.99 (0.80, 1.23) 0.942 | 41.6 | 0.98 (0.88, 1.08) 0.113 | 0.0 |
| S2 | 7 | 0.94 (0.83, 1.06) 0.336 | 73.6 | 0.86 (0.64, 1.16) 0.312 | 80.8 | 0.99 (0.84, 1.17) 0.907 | 61.0 | 0.96 (0.81, 1.14) 0.626 | 66.7 | 1.16 (0.96, 1.14) 0.634 | 78.6 |
| Cancer type |  |  |  |  |  |  |  |  |  |  |  |
| BC | 3 | 0.85 (0.75, 0.95) 0.004^*^ | 0.0 | 0.72 (0.57, 0.92) 0.007^*^ | 0.0 | 0.74 (0.61, 0.90) 0.002^*^ | 0.0 | 0.74 (0.61, 0.88) 0.001^*^ | 0.0 | 0.89 (0.73, 1.08) 0.223 | 0.0 |
| EC | 3 | 1.04 (0.96, 1.13) 0.292 | 0.0 | 1.09 (0.93, 1.28) 0.270 | 0.0 | 1.17 (1.02, 1.34) 0.028^*^ | 0.0 | 1.14 (1.00, 1.30) 0.047^*^ | 0.0 | 0.98 (0.87, 1.12) 0.789 | 0.0 |
| NSCLC | 1 | 1.16 (0.94, 1.43) 0.161 | _ | 1.56 (0.93, 2.60) 0.090 | _ | 1.04 (0.80, 1.35) 0.748 | _ | 1.11 (0.87, 1.42) 0.401 | _ | 1.54 (0.93, 2.55) 0.094 | _ |
| GC | 1 | 1.13 (0.94, 1.37) 0.206 | _ | 1.28 (0.87, 1.87) 0.207 | _ | 1.25 (0.90, 1.74) 0.188 | _ | 1.26 (0.92, 1.72) 0.150 | _ | 1.10 (0.81, 1.50) 0.540 | _ |
| OC | 1 | 1.55 (1.09, 2.21) 0.016^*^ | _ | 1.97 (0.88, 4.43) 0.100 | _ | 1.60 (1.00, 2.56) 0.053 | _ | 1.67 (1.07, 2.59) 0.023^*^ | _ | 1.67 (0.76, 3.69) 0.203 | _ |
| CRC | 3 | 1.08 (0.91, 1.27) 0.380 | 0.0 | 1.16 (0.81, 1.66) 0.425 | 0.0 | 1.00 (0.76, 1.32) 0.997 | 0.0 | 1.05 (0.80, 1.36) 0.735 | 0.0 | 1.19 (0.89, 1.59) 0.250 | 0.0 |
| Melanoma | 1 | 1.00 (0.57, 1.77) 1.000 | _ | 4.96 (0.24, 103.81) 0.302 | _ | 0.83 (0.45, 1.53) 0.555 | _ | 0.91 (0.50, 1.65) 0.761 | _ | 5.04 (0.24, 105.52) 0.297 | _ |
| HNSCC | 1 | 1.00 (0.25, 4.04) 1.000 | _ | _ | _ | 1.00 (0.25, 4.08) 1.000 | _ | 1.00 (0.25, 1.07) 1.000 | _ | _ | _ |
| Myeloma | 1 | 0.88 (0.64, 1.20) 0.407 | _ | 0.83 (0.44, 1.57) 0.569 | _ | 0.61 (0.36, 1.01) 0.056 | _ | 0.66 (0.41, 1.07) 0.095 | _ | 1.13 (0.65, 1.97) 0.655 | _ |
| Leukemia | 1 | 1.59 (0.30, 1.14) 0.117 | _ | 0.67 (0.10, 4.39) 0.677 | _ | 0.42 (0.18, 1.00) 0.051 | _ | 0.45 (0.20, 1.03) 0.058 | _ | 0.96 (0.15, 6.06) 0.969 | _ |
| AML | 1 | 0.66 (0.53, 0.83) 0.000^*^ | _ | 0.27 (0.15, 0.48) 0.000^*^ | _ | 1.04 (0.72, 1.50) 0.847 | _ | 0.80 (0.56, 1.13) 0.205 | _ | 0.26 (0.16, 0.44) 0.000^*^ | _ |
| PD-1.3 rs11568821G/A | | | | | | | | | | | |
| Ethnicity |  |  |  |  |  |  |  |  |  |  |  |
| Caucasian | 2 | 2.72 (0.33, 22.72) 0.356 | 75.9 | 2.36 (0.74, 7.52) 0.146 | 0.0 | 1.16 (0.74, 1.83) 0.524 | 59.2 | 2.37 (0.33, 17.10) 0.393 | 70.9 | 2.28 (0.71, 7.27) 0.165 | 0.0 |
| Asian | 11 | 0.88 (0.69, 1.14) 0.339 | 70.2 | 1.23 (0.81, 1.87) 0.338 | 46.3 | 0.80 (0.69, 0.93) 0.004^*^ | 32.0 | 0.85 (0.67, 1.08) 0.182 | 56.0 | 1.26 (0.84, 1.88) 0.260 | 31.1 |
| Source of control |  |  |  |  |  |  |  |  |  |  |  |
| PB | 6 | 0.83 (0.69, 0.99) 0.042^*^ | 15.0 | 0.97 (0.52, 1.79) 0.921 | 0.0 | 0.79 (0.66, 0.94) 0.009^*^ | 0.0 | 0.80 (0.67, 0.95) 0.012^*^ | 0.0 | 1.01 (0.55, 1.86) 0.979 | 0.0 |
| HB | 7 | 1.12 (0.69, 1.82) 0.657 | 81.0 | 1.66 (0.99, 2.78) 0.053 | 65.5 | 0.92 (0.73, 1.16) 0.468 | 59.0 | 1.09 (0.68, 1.77) 0.714 | 73.7 | 1.60 (0.99, 5.58) 0.053 | 53.2 |
| NOS scores |  |  |  |  |  |  |  |  |  |  |  |
| N1 | 8 | 1.03 (0.66, 1.59) 0.908 | 79.9 | 1.51 (0.93, 2.45) 0.096 | 61.0 | 0.86 (0.70, 1.07) 0.188 | 57.7 | 1.00 (0.65, 1.53) 0.985 | 72.2 | 1.49 (0.95, 2.34) 0.086 | 47.3 |
| N2 | 5 | 0.86 (0.71, 1.03) 0.106 | 13.2 | 1.04 (0.53, 2.05) 0.901 | 0.0 | 0.81 (0.67, 0.98) 0.030^*^ | 0.0 | 0.82 (0.69, 0.99) 0.039^*^ | 0.0 | 1.07 (0.55, 2.11) 0.835 | 0.0 |
| Sample size |  |  |  |  |  |  |  |  |  |  |  |
| S1 | 12 | 0.96 (0.72, 1.27) 0.556 | 71.6 | 1.40 (0.94, 2.08) 0.102 | 38.7 | 0.86 (0.73, 1.01) 0.064 | 40.9 | 0.92 (0.71, 1.21) 0.563 | 60.7 | 1.40 (0.96, 2.06) 0.083 | 19.8 |
| S2 | 1 | 0.77 (0.59, 1.01) 0.058 | _ | 0.21 (0.01, 4.47) 0.320 | _ | 0.77 (0.58, 1.02) 0.072 | _ | 0.76 (0.57, 1.01) 0.058 | _ | 0.23 (0.01, 4.73) 0.338 | _ |
| Cancer type |  |  |  |  |  |  |  |  |  |  |  |
| BC | 3 | 1.59 (0.63, 4.04) 0.328 | 78.1 | 1.67 (0.55, 5.03) 0.364 | 0.0 | 1.00 (0.72, 1.39) 0.982 | 75.9 | 1.52 (0.58, 3.97) 0.391 | 77.8 | 1.69 (0.56, 5.12) 0.351 | 77.8 |
| HCC | 1 | 0.78 (0.52, 1.19) 0.248 | _ | _ | _ | 0.76 (0.49, 1.18) 0.218 | _ | 0.76 (0.49, 1.18) 0.218 | _ | _ | _ |
| CRC | 1 | 2.36 (1.54, 3.61) 0.000^*^ | _ | 3.80 (1.77, 8.18) 0.001^*^ | _ | 1.84 (0.88, 3.88) 0.108 | _ | 2.60 (1.35, 5.01) 0.004^*^ | _ | 2.76 (1.44, 5.27) 0.002^*^ | _ |
| NSCLC | 2 | 0.82 (0.65, 1.04) 0.106 | 0.0 | 0.73 (0.21, 2.61) 0.630 | 0.0 | 0.81 (0.63, 1.05) 0.109 | 0.0 | 0.81 (0.63, 1.04) 0.099 | 0.0 | 0.74 (0.21, 2.65) 0.648 | 0.0 |
| TC | 1 | 0.58 (0.30, 1.12) 0.106 | _ | 0.22 (0.01, 4.33) 0.320 | _ | 0.67 (0.33, 1.36) 0.269 | _ | 0.61 (0.30, 1.23) 0.166 | _ | 0.24 (0.01, 4.61) 0.341 | _ |
| Brain tumor | 1 | 0.62 (0.36, 1.05) 0.076 | _ | 0.67 (0.15, 3.05) 0.604 | _ | 0.57 (0.30, 1.06) 0.074 | _ | 0.58 (0.32, 1.04) 0.069 | _ | 0.74 (0.16, 3.34) 0.690 | _ |
| HNSCC | 1 | 0.81 (0.50, 1.31) 0.393 | _ | 0.76 (0.20, 2.90) 0.688 | _ | 0.80 (0.45, 1.42) 0.449 | _ | 0.80 (0.46, 1.37) 0.408 | _ | 0.80 (0.21, 3.02) 0.736 | _ |
| BCC | 1 | 0.61 (0.39, 0.95) 0.028^*^ | _ | 0.60 (0.15, 2.34) 0.459 | _ | 0.58 (0.35, 0.96) 0.035^*^ | _ | 0.58 (0.36, 0.94) 0.028^*^ | _ | 0.65 (0.17, 2.54) 0.533 | _ |
| Leukemia | 1 | 0.67 (0.34, 1.31) 0.242 | _ | 0.41 (0.09, 2.03) 0.277 | _ | 0.77 (0.31, 1.86) 0.556 | _ | 0.68 (0.30, 1.57) 0.369 | _ | 0.46 (0.10, 2.16) 0.322 | _ |
| RCC | 1 | 1.16 (0.77, 1.74) 0.480 | _ | 1.88 (0.52, 6.79) 0.333 | _ | 1.04 (0.65, 1.67) 0.879 | _ | 1.11 (0.70, 1.74) 0.665 | _ | 1.87 (0.52, 6.72) 0.337 | _ |
| PD-1.1 rs36084323G/A | | | | | | | | | | | |
| Ethnicity |  |  |  |  |  |  |  |  |  |  |  |
| Caucasian | 3 | 0.62 (0.16, 2.38) 0.483 | 88.2 | 12.94 (0.73, 231.10) 0.082 | _ | 0.46 (0.12, 1.76) 0.253 | 86.2 | 0.51 (0.12, 2.14) 0.112 | 88.4 | 13.32 (0.75, 237.70) 0.078 | _ |
| Asian | 12 | 0.97 (0.82, 1.14) 0.688 | 86.0 | 1.12 (0.88, 1.44) 0.352 | 69.4 | 0.97 (0.81, 1.17) 0.771 | 69.6 | 0.96 (0.77, 1.20) 0.305 | 81.5 | 1.11 (0.93, 1.32) 0.245 | 59.0 |
| Source of control |  |  |  |  |  |  |  |  |  |  |  |
| PB | 7 | 1.00 (0.90, 1.11) 0.966 | 23.2 | 1.06 (0.84, 1.33) 0.629 | 25.7 | 0.94 (0.77, 1.14) 0.534 | 47.5 | 0.97 (0.81, 1.16) 0.742 | 42.8 | 1.04 (0.90, 1.21) 0.586 | 0.0 |
| HB | 8 | 0.78 (0.56, 1.09) 0.147 | 92.1 | 1.17 (0.74, 1.85) 0.507 | 81.7 | 0.78 (0.53, 1.16) 0.218 | 85.1 | 0.74 (0.48, 1.16) 0.188 | 90.1 | 1.16 (0.82, 1.64) 0.406 | 77.2 |
| NOS scores |  |  |  |  |  |  |  |  |  |  |  |
| N1 | 9 | 0.81 (0.60, 1.09) 0.161 | 91.1 | 1.14 (0.76, 1.71) 0.534 | 78.7 | 0.77 (0.54, 1.11) 0.160 | 83.8 | 0.75 (0.50, 1.12) 0.158 | 88.9 | 1.17 (0.87, 1.53) 0.311 | 72.6 |
| N2 | 6 | 1.00 (0.89, 1.13) 0.961 | 34.9 | 1.08 (0.82, 1.42) 0.574 | 38.2 | 0.97 (0.79, 1.19) 0.793 | 49.2 | 1.00 (0.83, 1.21) 0.982 | 47.0 | 1.03 (0.87, 1.22) 0.714 | 7.3 |
| Sample size |  |  |  |  |  |  |  |  |  |  |  |
| S1 | 9 | 0.74 (0.46, 1.19) 0.209 | 90.7 | 0.99 (0.42, 2.32) 0.975 | 83.3 | 0.69 (0.42, 1.11) 0.126 | 82.0 | 0.68 (0.39, 1.19) 0.177 | 88.0 | 1.10 (0.59, 2.05) 0.772 | 76.1 |
| S2 | 6 | 1.04 (0.95, 1.15) 0.408 | 56.1 | 1.15 (0.99, 1.35) 0.074 | 23.0 | 1.04 (0.88, 1.21) 0.671 | 55.4 | 1.05 (0.90, 1.23) 0.509 | 59.1 | 1.09 (0.96, 1.23) 0.192 | 20.7 |
| Cancer type |  |  |  |  |  |  |  |  |  |  |  |
| BC | 1 | 1.18 (0.99, 1.40) 0.069 | _ | 1.41 (0.98, 2.03) 0.065 | _ | 1.42 (1.04, 1.93) 0.026^*^ | _ | 1.41 (1.06, 1.89) 0.020^*^ | _ | 1.11 (0.82, 1.49) 0.497 | _ |
| NSCLC | 1 | 1.00 (0.85, 1.19) 0.963 | _ | 1.01 (0.73, 1.40) 0.951 | _ | 0.90 (0.68, 1.19) 0.467 | _ | 0.94 (0.72, 1.22) 0.629 | _ | 1.08 (0.83, 1.41) 0.571 | _ |
| EC | 3 | 0.97 (0.83, 1.12) 0.639 | 61.8 | 1.06 (0.89, 1.27) 0.508 | 0.0 | 0.99 (0.78, 1.25) 0.913 | 67.2 | 0.98 (0.77, 1.24) 0.843 | 68.9 | 0.99 (0.86, 1.15) 0.919 | 0.0 |
| OC | 2 | 1.38 (0.99, 1.91) 0.059 | 64.7 | 1.88 (0.99, 3.56) 0.053 | 61.8 | 1.24 (0.77, 2.01) 0.378 | 52.9 | 1.45 (0.84, 2.52) 0.185 | 65.9 | 1.50 (1.18, 1.91) 0.001^*^ | 0.0 |
| CRC | 3 | 0.26 (0.04, 1.58) 0.144 | 97.2 | 0.13 (0.00, 17.17) 0.410 | 95.4 | 0.27 (0.06, 1.34) 0.110 | 94.0 | 0.22 (0.03, 1.50) 0.122 | 96.1 | 0.18 (0.00, 10.91) 0.416 | 93.7 |
| Melanoma | 1 | 1.21 (0.70, 2.09) 0.489 | _ | 12.94 (0.73, 231.10) 0.082 | _ | 0.72 (0.38, 1.35) 0.303 | _ | 0.96 (0.53, 1.72) 0.880 | _ | 13.32 (0.75, 237.70) 0.078 | _ |
| Myeloma | 1 | 0.95 (0.69, 1.30) 0.731 | _ | 0.91 (0.49, 1.68) 0.761 | _ | 0.68 (0.40, 1.15) 0.152 | _ | 0.75 (0.46, 1.23) 0.256 | _ | 1.17 (0.70, 1.94) 0.551 | _ |
| HCC | 1 | 0.99 (0.45, 2.18) 0.985 | _ | _ | _ | 0.99 (0.44, 2.23) 0.984 | _ | 0.99 (0.44, 2.23) 0.984 | _ | _ | _ |
| RCC | 1 | 1.24 (0.43, 3.55) 0.695 | _ | _ | _ | 1.23 (0.43, 3.59) 0.693 | _ | 1.24 (0.43, 3.59) 0.693 | _ | _ | _ |
| BCC | 1 | 1.16 (0.80, 1.68) 0.445 | _ | 1.23 (0.46, 3.27) 0.679 | _ | 1.16 (0.74, 1.83) 0.517 | _ | 1.72 (0.76, 1.80) 0.468 | _ | 1.19 (0.45, 3.14) 0.730 | _ |
| PD-1 rs7421861T/C | | | | | | | | | | | |
| Ethnicity |  |  |  |  |  |  |  |  |  |  |  |
| Caucasian | 1 | 0.93 (0.71, 1.21) 0.578 | _ | 1.14 (0.64, 2.05) 0.656 | _ | 0.65 (0.44, 0.96) 0.031^*^ | _ | 0.74 (0.51, 1.07) 0.105 | _ | 1.43 (0.83, 2.47) 0.204 | _ |
| Asian | 7 | 1.04 (0.89, 1.20) 0.646 | 76.9 | 0.87 (0.72, 1.06) 0.171 | 23.6 | 1.15 (1.05, 1.26) 0.193 | 61.9 | 1.07 (0.91, 1.27) 0.421 | 70.7 | 0.86 (0.74, 1.00) 0.044^*^ | 0.0 |
| Source of control |  |  |  |  |  |  |  |  |  |  |  |
| PB | 4 | 0.94 (0.82, 1.09) 0.431 | 49.5 | 0.80 (0.63, 1.02) 0.072 | 0.0 | 0.92 (0.72, 1.18) 0.520 | 62.7 | 0.92 (0.73, 1.15) 0.449 | 59.9 | 0.86 (0.73, 1.01) 0.068 | 22.1 |
| HB | 4 | 1.10 (0.92, 1.32) 0.292 | 69.6 | 1.05 (0.79, 1.41) 0.722 | 20.3 | 1.18 (0.97, 1.43) 0.095 | 63.0 | 1.15 (0.94, 1.42) 0.174 | 69.2 | 0.99 (0.74, 1.32) 0.943 | 0.0 |
| NOS scores |  |  |  |  |  |  |  |  |  |  |  |
| N1 | 4 | 1.10 (0.92, 1.32) 0.292 | 69.6 | 1.05 (0.79, 1.41) 0.722 | 20.3 | 1.18 (0.97, 1.43) 0.095 | 63.0 | 1.15 (0.94, 1.42) 0.174 | 69.2 | 0.99 (0.74, 1.32) 0.943 | 0.0 |
| N2 | 4 | 0.94 (0.82, 1.09) 0.431 | 49.5 | 0.80 (0.63, 1.02) 0.072 | 0.0 | 0.92 (0.72, 1.18) 0.520 | 62.7 | 0.92 (0.73, 1.15) 0.449 | 59.9 | 0.86 (0.73, 1.01) 0.068 | 22.1 |
| Sample size |  |  |  |  |  |  |  |  |  |  |  |
| S1 | 2 | 0.91 (0.76, 1.10) 0.325 | 0.0 | 0.91 (0.56, 1.46) 0.685 | 40.0 | 0.81 (0.54, 1.21) 0.310 | 62.1 | 0.85 (0.68, 1.07) 0.168 | 0.0 | 1.08 (0.69, 1.68) 0.754 | 66.8 |
| S2 | 6 | 1.06 (0.90, 1.25) 0.511 | 79.7 | 0.90 (0.73, 1.10) 0.277 | 27.5 | 1.13 (0.95, 1.34) 0.171 | 65.5 | 1.09 (0.91, 1.32) 0.347 | 73.2 | 0.87 (0.74, 1.01) 0.071 | 0.0 |
| Cancer type |  |  |  |  |  |  |  |  |  |  |  |
| BC | 2 | 1.04 (0.85, 1.28) 0.692 | 42.1 | 0.89 (0.56, 1.43) 0.629 | 0.0 | 1.09 (0.86, 1.39) 0.478 | 41.8 | 1.07 (0.84, 1.37) 0.565 | 46.1 | 0.88 (0.55, 1.40) 0.586 | 0.0 |
| EC | 3 | 1.03 (0.76, 1.39) 0.844 | 90.9 | 0.89 (0.71, 1.13) 0.343 | 70.3 | 1.08 (0.78, 1.51) 0.652 | 82.7 | 1.04 (0.72, 1.51) 0.836 | 87.3 | 0.87 (0.74, 1.03) 0.095 | 42.0 |
| CRC | 1 | 1.17 (0.94, 1.44) 0.158 | _ | 0.92 (0.45, 1.89) 0.814 | _ | 1.28 (1.00, 1.64) 0.055 | _ | 1.24 (0.98, 1.59) 0.078 | _ | 0.85 (0.42, 1.73) 0.664 | _ |
| GC | 1 | 0.90 (0.70, 1.16) 0.408 | _ | 0.57 (0.24, 1.37) 0.210 | _ | 0.98 (0.72, 1.32) 0.885 | _ | 0.93 (0.70, 1.25) 0.633 | _ | 0.58 (0.24, 1.37) 0.213 | _ |
| RCC | 1 | 0.93 (0.71, 1.21) 0.578 | _ | 1.14 (0.64, 2.05) 0.656 | _ | 0.65 (0.44, 1.51) 0.031 | _ | 0.74 (0.51, 1.07) 0.105 | _ | 1.43 (0.83, 2.47) 0.204 | _ |
| PD-L1 rs4143815G/C | | | | | | | | | | | |
| Ethnicity |  |  |  |  |  |  |  |  |  |  |  |
| Caucasian | 2 | 0.99 (0.89, 1.10) 0.832 | 0.0 | 0.96 (0.75, 1.22) 0.731 | 0.0 | 0.99 (0.85, 1.17) 0.961 | 0.0 | 0.99 (0.85, 1.16) 0.910 | 0.0 | 0.97 (0.79, 1.20) 0.804 | 0.0 |
| Asian | 10 | 0.83 (0.67, 1.03) 0.083 | 85.5 | 0.70 (0.46, 1.07) 0.096 | 84.2 | 0.75 (0.52, 1.08) 0.126 | 82.0 | 0.73 (0.50, 1.07) 0.103 | 84.9 | 0.84 (0.66, 1.06) 0.144 | 72.7 |
| Source of control |  |  |  |  |  |  |  |  |  |  |  |
| PB | 4 | 0.93 (0.75, 1.15) 0.490 | 63.5 | 0.84 (0.55, 1.27) 0.401 | 58.8 | 0.87 (0.69, 1.11) 0.260 | 0.0 | 0.87 (0.70, 1.09) 0.218 | 0.0 | 0.94 (0.67, 1.33) 0.737 | 68.3 |
| HB | 8 | 0.82 (0.65, 1.04) 0.098 | 88.1 | 0.69 (0.43, 1.11) 0.127 | 87.0 | 0.75 (0.49, 1.14) 0.178 | 87.8 | 0.73 (0.47, 1.12) 0.149 | 90.0 | 0.82 (0.65, 1.04) 0.104 | 68.0 |
| NOS scores |  |  |  |  |  |  |  |  |  |  |  |
| N1 | 8 | 0.84 (0.66, 1.06) 0.144 | 88.6 | 0.74 (0.46, 1.20) 0.221 | 87.7 | 0.80 (0.53, 1.20) 0.277 | 87.3 | 0.77 (0.51, 1.18) 0.238 | 89.9 | 0.83 (0.65, 1.06) 0.142 | 70.2 |
| N2 | 4 | 0.90 (0.73, 1.09) 0.275 | 5533 | 0.75 (0.51, 1.03) 0.140 | 49.1 | 0.81 (0.64, 1.04) 0.102 | 1.8 | 0.80 (0.63, 1.02) 0.075 | 7.2 | 0.93 (0.67, 1.28) 0.635 | 64.2 |
| Sample size |  |  |  |  |  |  |  |  |  |  |  |
| S1 | 10 | 0.82 (0.66, 1.01) 0.066 | 83.6 | 0.68 (0.44, 1.04) 0.077 | 82.3 | 0.74 (0.51, 1.08) 0.122 | 81.8 | 0.72 (0.49, 1.05) 0.091 | 83.6 | 0.82 (0.65, 1.04) 0.107 | 68.9 |
| S2 | 2 | 1.01 (0.91, 1.11) 0.885 | 0.0 | 1.00 (0.81, 1.23) 0.977 | 0.0 | 0.99 (0.86, 1.16) 0.950 | 0.0 | 1.00 (0.87, 1.15) 0.979 | 0.0 | 1.03 (0.86, 1.22) 0.771 | 0.0 |
| Cancer type |  |  |  |  |  |  |  |  |  |  |  |
| GC | 3 | 0.66 (0.45, 0.99) 0.045^*^ | 85.7 | 0.44 (0.24, 0.81) 0.008^*^ | 74.8 | 0.51 (0.29, 0.92) 0.025^*^ | 74.8 | 0.49 (0.28, 0.85) 0.012^*^ | 74.8 | 0.69 (0.43, 1.12) 0.136 | 74.8 |
| NSCLC | 1 | 1.06 (0.82, 1.36) 0.678 | _ | 1.20 (0.73, 1.97) 0.479 | _ | 1.39 (0.85, 2.29) 0.188 | _ | 1.30 (0.82, 2.05) 0.265 | _ | 0.95 (0.66, 1.36) 0.775 | _ |
| EC | 1 | 1.02 (0.87, 1.21) 0.784 | _ | 1.02 (0.71, 1.45) 0.932 | _ | 0.94 (0.67, 1.32) 0.706 | _ | 0.97 (0.70, 134) 0.849 | _ | 1.07 (0.84, 1.36) 0.592 | _ |
| OC | 1 | 0.65 (0.48, 0.88) 0.005^*^ | _ | 0.43 (0.23, 0.79) 0.006^*^ | _ | 0.78 (0.47, 1.32) 0.359 | _ | 0.64 (0.39, 1.04) 0.072 | _ | 0.50 (0.30, 0.83) 0.007^*^ | _ |
| HCC | 2 | 0.74 (0.44, 1.23) 0.245 | 81.5 | 0.46 (0.21, 0.99) 0.047^*^ | 63.5 | 0.42 (0.28, 0.64) 0.000^*^ | 63.5 | 0.43 (0.29, 0.63) 0.000^*^ | 63.5 | 0.86 (0.41, 1.84) 0.706 | 63.5 |
| CRC | 1 | 1.00 (0.89, 1.13) 0.985 | _ | 0.99 (0.76, 0.99) 0.921 | _ | 1.01 (0.85, 1.20) 0.907 | _ | 1.01 (0.86, 1.18) 0.949 | _ | 0.98 (0.76, 1.27) 0.888 | _ |
| Myeloma | 1 | 1.15 (0.84, 1.58) 0.388 | _ | 1.27 (0.67, 2.41) 0.469 | _ | 0.85 (0.48, 1.54) 0.599 | _ | 0.98 (0.57, 1.71) 0.955 | _ | 1.42 (0.87, 2.31) 0.160 | _ |
| BC | 1 | 1.14 (0.89, 1.46) 0.312 | _ | 1.93 (1.04, 3.57) 0.037^*^ | _ | 2.45 (1.37, 4.37) 0.003^*^ | _ | 2.25 (1.28, 3.95) 0.005^*^ | _ | 0.91 (0.63, 1.33) 0.636 | _ |
| RCC | 1 | 0.93 (0.71, 1.23) 0.621 | _ | 0.83 (0.45, 1.51) 0.532 | _ | 0.83 (0.45, 1.52) 0.091 | _ | 0.83 (0.47, 1.47) 0.514 | _ | 0.96 (0.66, 1.38) 0.816 | _ |
| PD-1.6 rs10204525A/G | | | | | | | | | | | |
| Ethnicity |  |  |  |  |  |  |  |  |  |  |  |
| Caucasian | 1 | 1.21 (0.78, 1.88) 0.400 | _ | 4.96 (0.59, 41.61) 0.140 | _ | 5.00 (0.57, 43.53) 0.145 | _ | 4.97 (0.59, 41.60) 0.139 | _ | 1.10 (0.68, 1.78) 0.688 | _ |
| Asian | 12 | 0.97 (0.85, 1.11) 0.647 | 77.3 | 0.96 (0.71, 1.28) 0.764 | 71.2 | 1.02 (0.88, 1.17) 0.839 | 61.7 | 1.00 (0.85, 1.18) 0.983 | 71.8 | 0.95 (0.77, 1.17) 0.596 | 60.8 |
| Source of control |  |  |  |  |  |  |  |  |  |  |  |
| PB | 5 | 0.94 (0.71, 1.24) 0.663 | 88.2 | 0.90 (0.46, 1.74) 0.749 | 84.1 | 0.96 (0.71, 1.30) 0.792 | 78.6 | 0.94 (0.66, 1.34) 0.739 | 85.8 | 0.88 (0.57, 1.37) 0.584 | 76.8 |
| HB | 8 | 1.02 (0.90, 1.15) 0.797 | 53.1 | 1.05 (0.77, 1.42) 0.769 | 53.2 | 1.06 (0.90, 1.25) 0.455 | 41.7 | 1.06 (0.90, 1.25) 0.462 | 45.3 | 0.99 (0.81, 1.21) 0.936 | 36.3 |
| NOS scores |  |  |  |  |  |  |  |  |  |  |  |
| N1 | 8 | 1.02 (0.90, 1.15) 0.797 | 53.1 | 1.05 (0.77, 1.42) 0.769 | 53.2 | 1.06 (0.90, 1.25) 0.455 | 41.7 | 1.06 (0.90, 1.25) 0.462 | 45.3 | 0.99 (0.81, 1.21) 0.936 | 36.3 |
| N2 | 5 | 0.94 (0.71, 1.24) 0.663 | 88.2 | 0.90 (0.46, 1.74) 0.749 | 84.1 | 0.96 (0.71, 1.30) 0.792 | 78.6 | 0.94 (0.66, 1.34) 0.739 | 85.8 | 0.88 (0.57, 1.37) 0.584 | 76.8 |
| Sample size |  |  |  |  |  |  |  |  |  |  |  |
| S1 | 7 | 0.88 (0.69, 1.13) 0.310 | 76.2 | 0.84 (0.43, 1.63) 0.597 | 71.7 | 0.91 (0.63, 1.33) 0.631 | 64.4 | 0.90 (0.60, 1.35) 0.602 | 72.3 | 0.87 (0.65, 1.16) 0.340 | 53.2 |
| S2 | 6 | 1.06 (0.93, 1.21) 0.380 | 72.5 | 1.09 (0.80, 1.48) 0.584 | 70.2 | 1.83 (0.95, 1.24) 0.253 | 53.6 | 1.09 (0.93, 1.26) 0.281 | 65.9 | 1.05 (0.80, 1.37) 0.743 | 64.0 |
| Cancer type |  |  |  |  |  |  |  |  |  |  |  |
| HCC | 2 | 0.79 (0.36, 1.74) 0.561 | 89.0 | 0.78 (0.08, 8.08) 0.838 | 88.5 | 1.11 (0.23, 5.33) 0.900 | 89.0 | 1.05 (0.20, 5.43) 0.954 | 82.6 | 0.61 (0.18, 2.04) 0.421 | 84.6 |
| EC | 4 | 1.00 (0.85, 1.18) 0.989 | 77.0 | 0.98 (0.65, 1.49) 0.925 | 79.2 | 1.02 (0.88, 1.17) 0.822 | 77.0 | 1.01 (0.86, 1.19) 0.895 | 65.0 | 0.98 (0.66, 1.44) 0.904 | 77.1 |
| GC | 1 | 0.92 (0.74, 1.15) 0.466 | _ | 0.72 (0.42, 1.24) 0.241 | _ | 1.03 (0.77, 1.37) 0.857 | _ | 0.97 (0.74, 1.28) 0.817 | _ | 0.72 (0.42, 1.21) 0.213 | _ |
| BC | 1 | 1.12 (0.94, 1.34) 0.202 | _ | 1.20 (0.79, 1.82) 0.395 | _ | 1.17 (0.92, 1.49) 0.208 | _ | 1.18 (0.93, 1.48) 0.174 | _ | 1.11 (0.75, 1.66) 0.600 | _ |
| CRC | 2 | 0.73 (0.31, 1.72) 0.467 | 86.9 | 0.32 (0.01, 9.62) 0.514 | 82.2 | 0.34 (0.02, 5.54) 0.450 | 86.9 | 0.31 (0.02, 6.34) 0.448 | 78.3 | 0.89 (0.40, 1.95) 0.766 | 73.1 |
| RCC | 1 | 1.21 (0.78, 1.88) 0.400 | _ | 4.96 (0.59, 41.61) 0.140 | _ | 5.00 (0.57, 43.53) 0.145 | _ | 4.97 (0.59, 41.60) 0.139 | _ | 1.10 (0.68, 1.78) 0.688 | _ |
| BCC | 1 | 1.00 (0.71, 1.42) 0.980 | _ | 0.88 (0.36, 2.13) 0.774 | _ | 0.82 (0.32, 2.08) 0.678 | _ | 0.86 (0.36, 2.07) 0.739 | _ | 1.04 (0.69, 1.56) 0.855 | _ |
| AML | 1 | 1.34 (1.06, 1.71) 0.017^*^ | _ | 1.63 (0.93, 2.85) 0.088 | _ | 1.49 (1.06, 2.09) 0.020^*^ | _ | 1.52 (1.10, 2.09) 0.011^*^ | _ | 1.34 (0.78, 2.28) 0.287 | _ |
| PD-L1 rs2890658A/C | | | | | | | | | | | |
| Ethnicity |  |  |  |  |  |  |  |  |  |  |  |
| Asian | 9 | 1.03 (0.75, 1.42) 0.859 | 85.2 | 0.91 (0.59, 1.41) 0.671 | 9.4 | 1.14 (0.82, 1.57) 0.441 | 67.4 | 1.07 (0.79, 1.56) 0.562 | 72.9 | 0.81 (0.50, 1.32) 0.400 | 57.3 |
| Source of control |  |  |  |  |  |  |  |  |  |  |  |
| PB | 2 | 1.13 (0.65, 1.97) 0.661 | 89.4 | 0.97 (0.53, 1.77) 0.909 | 24.0 | 1.34 (0.85, 2.11) 0.208 | 38.3 | 1.23 (0.67, 2.26) 0.503 | 63.3 | 0.90 (0.60, 1.37) 0.636 | 13.4 |
| HB | 7 | 1.00 (0.65, 1.54) 0.997 | 85.9 | 0.86 (0.46, 1.60) 0.623 | 19.4 | 1.08 (0.71, 1.65) 0.715 | 71.7 | 1.06 (0.68, 1.67) 0.787 | 76.1 | 0.76 (0.38, 1.51) 0.427 | 38.4 |
| NOS scores |  |  |  |  |  |  |  |  |  |  |  |
| N1 | 6 | 1.07 (0.65, 1.75) 0.790 | 87.9 | 0.99 (0.51, 1.92) 0.967 | 19.3 | 1.17 (0.73, 1.87) 0.521 | 72.7 | 1.16 (0.71, 1.90) 0.564 | 76.5 | 0.88 (0.40, 1.92) 0.739 | 47.4 |
| N2 | 3 | 0.98 (0.62, 1.54) 0.928 | 83.9 | 0.86 (0.48, 1.52) 0.600 | 22.7 | 1.06 (0.63, 1.81) 0.820 | 67.9 | 1.00 (0.55, 1.81) 0.995 | 75.6 | 0.87 (0.60, 1.25) 0.443 | 6.7 |
| Sample size |  |  |  |  |  |  |  |  |  |  |  |
| S1 | 6 | 0.95 (0.59, 1.54) 0.835 | 87.4 | 0.81 (0.42, 1.55) 0.525 | 0.525 | 1.02 (0.62, 1.69) 0.925 | 75.6 | 1.00 (0.59, 1.70) 0.999 | 79.5 | 0.72 (0.35, 1.51) 0.386 | 42.8 |
| S2 | 3 | 1.19 (0.75, 1.82) 0.410 | 80.7 | 1.00 (0.56, 1.80) 0.997 | 0.997 | 1.43 (1.10, 1.84) 0.007^*^ | 38.3 | 1.36 (0.99, 1.88) 0.062 | 26.6 | 0.87 (0.68, 1.12) 0.273 | 0.0 |
| Cancer type |  |  |  |  |  |  |  |  |  |  |  |
| NSCLC | 3 | 1.72 (1.39, 2.13) 0.000^*^ | 0.0 | 2.57 (0.97, 6.81) 0.057 | 0.0 | 1.74 (1.37, 2.19) 0.000^*^ | 0.0 | 0.64 (0.35, 1.15) 0.000^*^ | 0.0 | 2.29 (0.85, 6.16) 0.101 | 0.0 |
| GC | 1 | 0.67 (0.40, 1.14) 0.144 | _ | 0.62 (0.11, 3.47) 0.587 | _ | 0.64 (0.35, 1.18) 0.150 | _ | 0.83 (0.41, 1.66) 0.133 | _ | 0.69 (0.12, 3.85) 0.674 | _ |
| EC | 1 | 0.86 (0.69, 1.07) 0.180 | _ | 0.79 (0.39, 1.59) 0.507 | _ | 0.93 (0.45, 1.92) 0.848 | _ | 0.71 (0.51, 1.00) 0.590 | _ | 0.84 (0.65, 1.09) 0.182 | _ |
| HCC | 2 | 0.74 (0.55, 1.00) 0.046^*^ | 0.0 | 0.61 (0.23, 1.63) 0.323 | 0.0 | 0.72 (0.51, 1.03) 0.069 | 0.0 | 0.25 (0.03, 2.23) 0.049 | 0.0 | 0.69 (0.25, 1.92) 0.481 | 0.0 |
| BC | 1 | 0.53 (0.40, 0.71) 0.000^*^ | _ | 0.16 (0.02, 1.44) 0.102 | _ | 0.39 (0.04, 3.52) 0.400 | _ | 0.64 (0.35, 1.15) 0.213 | _ | 0.40 (0.28, 0.57) 0.000^*^ | _ |
| UCC | 1 | 1.40 (0.83, 2.36) 0.859 | _ | 1.72 (0.15, 19.07) 0.660 | _ | 1.40 (0.80, 2.47) 0.240 | _ | 1.42 (0.82, 2.47) 0.217 | _ | 1.65 (0.15, 18.28) 0.685 | _ |
| PD-L1 rs10815225G/C | | | | | | | | | | | |
| Ethnicity |  |  |  |  |  |  |  |  |  |  |  |
| Caucasian | 2 | 1.16 (0.99, 1.37) 0.061 | 0.0 | 0.69 (0.32, 1.45) 0.323 | 25.3 | 1.28 (1.03, 1.58) 0.024^*^ | 11.7 | 1.23 (1.03, 1.46) 0.211 | 0.0 | 0.65 (0.31, 1.37) 0.260 | 28.5 |
| Asian | 1 | 0.65 (0.45, 0.96) 0.028^*^ | _ | 6.63 (0.32, 138.52) 0.223 | _ | 0.57 (0.38, 0.85) 0.006^*^ | _ | 0.60 (0.40, 0.89) 0.011^*^ | _ | 7.18 (0.34, 150.03) 0.204 | _ |
| Source of control |  |  |  |  |  |  |  |  |  |  |  |
| PB | 1 | 1.26 (0.80, 1.97) 0.321 | _ | 0.15 (0.01, 2.73) 0.198 | _ | 1.62 (0.99, 2.66) 0.056 | _ | 1.45 (0.89, 2.35) 0.131 | _ | 0.13 (0.01, 2.51) 0.179 | _ |
| HB | 2 | 0.89 (0.51, 1.55) 0.682 | 85.9 | 1.03 (0.49, 2.20) 0.930 | 40.1 | 0.85 (0.40, 1.79) 0.666 | 91.0 | 0.87 (0.44, 1.72) 0.677 | 89.6 | 1.00 (0.47, 2.13) 0.996 | 46.9 |
| NOS scores |  |  |  |  |  |  |  |  |  |  |  |
| N1 | 2 | 0.89 (0.51, 1.55) 0.682 | 85.9 | 1.03 (0.49, 2.20) 0.930 | 40.1 | 0.85 (0.40, 1.79) 0.666 | 91.0 | 0.87 (0.44, 1.72) 0.677 | 89.6 | 1.00 (0.47, 2.13) 0.996 | 46.9 |
| N2 | 1 | 1.26 (0.80, 1.97) 0.321 | _ | 0.15 (0.01, 2.73) 0.198 | _ | 1.62 (0.99, 2.66) 0.056 | _ | 1.45 (0.89, 2.35) 0.131 | _ | 0.13 (0.01, 2.51) 0.179 | _ |
| Sample size |  |  |  |  |  |  |  |  |  |  |  |
| S1 | 2 | 0.90 (0.47, 1.70) 0.735 | 78.9 | 0.80 (0.19, 3.34) 0.755 | 68.3 | 0.95 (0.34, 2.66) 0.920 | 90.3 | 0.92 (0.39, 2.20) 0.851 | 87.0 | 0.78 (0.19, 3.23) 0.736 | 70.9 |
| S2 | 1 | 1.15 (0.97, 1.37) 0.104 | _ | 0.85 (0.38, 1.90) 0.687 | _ | 1.22 (1.01, 1.47) 0.043 | _ | 1.20 (0.99, 1.44) 0.057 | _ | 0.81 (0.36, 1.81) 0.606 | _ |
| Cancer type |  |  |  |  |  |  |  |  |  |  |  |
| GC | 1 | 0.65 (0.45, 0.96) 0.028^*^ | _ | 6.63 (0.32, 138.52) 0.223 | _ | 0.57 (0.38, 0.85) 0.006^*^ | _ | 0.60 (0.40, 0.89) 0.011^*^ | _ | 7.18 (0.34, 150.03) 0.204 | _ |
| CRC | 1 | 1.15 (0.97, 1.37) 0.104 | _ | 0.85 (0.38, 1.90) 0.687 | _ | 1.22 (1.01, 1.47) 0.043^*^ | _ | 1.20 (0.99, 1.44) 0.057 | _ | 0.81 (0.36, 1.81) 0.606 | _ |
| RCC | 1 | 1.26 (0.80, 1.97) 0.321 | _ | 0.15 (0.01, 2.73) 0.198 | _ | 1.62 (0.99, 2.66) 0.056 | _ | 1.45 (0.89, 2.35) 0.131 | _ | 0.13 (0.01, 2.51) 0.179 | _ |
| PD-L1 rs17718883C/G | | | | | | | | | | | |
| Ethnicity |  |  |  |  |  |  |  |  |  |  |  |
| Asian | 3 | 0.07 (0.20, 0.25) 0.000^*^ | 85.1 | 0.04 (0.01, 0.12) 0.000^*^ | 0.0 | 0.07 (0.02, 0.30) 0.000^*^ | 84.4 | 0.06 (0.02, 0.24) 0.000^*^ | 84.6 | 0.06 (0.02, 0.20) 0.000^*^ | 0.0 |
| Source of control |  |  |  |  |  |  |  |  |  |  |  |
| HB | 3 | 0.07 (0.20, 0.25) 0.000^*^ | 85.1 | 0.04 (0.01, 0.12) 0.000^*^ | 0.0 | 0.07 (0.02, 0.30) 0.000^*^ | 84.4 | 0.06 (0.02, 0.24) 0.000^*^ | 84.6 | 0.06 (0.02, 0.20) 0.000^*^ | 0.0 |
| NOS scores |  |  |  |  |  |  |  |  |  |  |  |
| N1 | 2 | 0.12 (0.04, 0.35) 0.000^*^ | 84.5 | 0.05 (0.01, 0.16) 0.000^*^ | 0.0 | 0.12 (0.03, 0.48) 0.003^*^ | 86.4 | 0.10 (0.03, 0.36) 0.000^*^ | 85.4 | 0.07 (0.02, 0.24) 0.000^*^ | 0.0 |
| N2 | 1 | 0.01 (0.00, 0.08) 0.000^*^ | _ | 0.02 (0.00, 0.32) 0.006^*^ | _ | 0.01 (0.00, 0.10) 0.928 | _ | 0.98 (0.62, 1.54) 0.928 | _ | 0.03 (0.00, 0.52) 0.928 | _ |
| Sample size |  |  |  |  |  |  |  |  |  |  |  |
| S1 | 3 | 0.07 (0.20, 0.25) 0.000^*^ | 85.1 | 0.04 (0.01, 0.12) 0.000^*^ | 0.0 | 0.07 (0.02, 0.30) 0.000^*^ | 84.4 | 0.06 (0.02, 0.24) 0.000^*^ | 84.6 | 0.06 (0.02, 0.20) 0.000^*^ | 0.0 |
| Cancer type |  |  |  |  |  |  |  |  |  |  |  |
| HCC | 2 | 0.03 (0.01, 0.23) 0.001^*^ | 73.5 | 0.03 (0.01, 1.13) 0.000^*^ | 0.0 | 0.04 (0.01, 0.15) 0.000^*^ | 51.5 | 0.03 (0.01, 0.16) 0.000^*^ | 65.3 | 0.05 (0.02, 0.20) 0.000^*^ | 0.0 |
| GC | 1 | 0.20 (0.11, 0.36) 0.000^*^ | _ | 0.06 (0.01, 0.43) 0.005^*^ | _ | 0.24 (0.12, 0.48) 0.000^*^ | _ | 0.19 (0.10, 0.37) 0.000^*^ | _ | 0.08 (0.01, 0.60) 0.014^*^ | _ |
| PD-L1 rs2297136G/C | | | | | | | | | | | |
| Ethnicity |  |  |  |  |  |  |  |  |  |  |  |
| Asian | 4 | 1.00 (0.75, 1.35) 0.982 | 71.4 | 0.85 (0.45, 1.62) 0.624 | 58.5 | 1.04 (0.57, 1.90) 0.889 | 83.7 | 1.03 (0.59, 1.41) 0.924 | 82.8 | 0.86 (0.51, 1.44) 0.554 | 55.7 |
| Source of control |  |  |  |  |  |  |  |  |  |  |  |
| PB | 1 | 0.95 (0.65, 1.39) 0.785 | _ | 1.14 (0.42, 3.12) 0.799 | _ | 0.86 (0.53, 1.39) 0.532 | _ | 0.89 (0.57, 1.41) 0.623 | _ | 1.20 (0.45, 3.24) 0.716 | _ |
| HB | 3 | 1.02 (0.69, 1.50) 0.931 | 80.6 | 0.79 (0.34, 1.81) 0.571 | 70.7 | 1.01 (0.50, 2.43) 0.816 | 87.3 | 1.06 (0.50, 2.23) 0.682 | 87.0 | 0.80 (0.42, 1.50) 0.484 | 67.1 |
| NOS scores |  |  |  |  |  |  |  |  |  |  |  |
| N1 | 3 | 0.95 (0.63, 1.42) 0.804 | 79.4 | 0.65 (0.36, 1.19) 0.163 | 31.1 | 1.03 (0.42, 2.50) 0.950 | 88.3 | 0.97 (0.41, 2.27) 0.942 | 88.3 | 0.69 (0.46, 1.05) 0.083 | 19.8 |
| N2 | 1 | 1.17 (0.86, 1.59) 0.331 | _ | 1.62 (0.78, 3.39) 0.196 | _ | 1.01 (0.68, 1.50) 0.975 | _ | 1.10 (0.76, 1.59) 0.614 | _ | 1.62 (0.78, 3.36) 0.194 | _ |
| Sample size |  |  |  |  |  |  |  |  |  |  |  |
| S1 | 4 | 1.00 (0.75, 1.35) 0.982 | 71.4 | 0.85 (0.45, 1.62) 0.624 | 58.5 | 1.04 (0.57, 1.90) 0.889 | 83.7 | 1.03 (0.59, 1.41) 0.924 |  | 0.86 (0.51, 1.44) 0.554 | 55.7 |
| Cancer type |  |  |  |  |  |  |  |  |  |  |  |
| GC | 1 | 1.17 (0.86, 1.59) 0.331 | _ | 1.62 (0.78, 3.39) 0.196 | _ | 1.01 (0.68, 1.50) 0.975 | _ | 1.10 (0.76, 1.59) 0.614 | _ | 1.62 (0.78, 3.36) 0.194 | _ |
| NSCLC | 1 | 1.30 (1.00, 1.70) 0.048^*^ | _ | 0.74 (0.32, 1.71) 0.484 | _ | 2.29 (1.56, 3.36) 0.000^*^ | _ | 2.09 (1.43, 3.04) 0.000^*^ | _ | 0.44 (0.20, 0.97) 0.042^*^ | _ |
| HCC | 1 | 0.68 (0.49, 0.93) 0.017^*^ | _ | 0.39 (0.18, 0.85) 0.018^*^ | _ | 0.48 (0.21, 1.07) 0.073 | _ | 0.42 (0.20, 0.90) 0.026^*^ | _ | 0.72 (0.48, 1.06) 0.094 | _ |
| Myeloma | 1 | 0.95 (0.65, 1.39) 0.785 | _ | 1.14 (0.42, 3.12) 0.799 | _ | 0.86 (0.53, 1.39) 0.532 | _ | 0.89 (0.57, 1.41) 0.623 | _ | 1.20 (0.45, 3.24) 0.716 | _ |

CC: cervical cancer, BC: breast cancer, HCC: hepatocellular carcinoma, CRC: colorectal cancer, HNSCC: head and neck squamous cell carcinomas, TC: thyroid cancer, EC: esophageal cancer, OC: ovarian cancer, NSCLC: non-small cell lung cancer, RCC: renal cell carcinoma, BCC: basal cell carcinoma; AML: acute myelocytic leukemia; UCC: urothelial cell carcinoma. ^*^*P*<0.05.
